# Wearable Sensors in Industrial Ergonomics: Enhancing Safety and Productivity in Industry 4.0

**DOI:** 10.3390/s25051526

**Published:** 2025-02-28

**Authors:** Jose E. Naranjo, Carlos A. Mora, Diego Fernando Bustamante Villagómez, María Gabriela Mancheno Falconi, Marcelo V. Garcia

**Affiliations:** 1 Facultad de Ciencias de la Ingeniería y Aplicadas, Universidad Técnica de Cotopaxi (UTC), Campus La Matriz, Ave. Simón Rodríguez, Latacunga 050102, Ecuador; carlos.mora7340@utc.edu.ec; 2 GSyA Research Group, Universidad de Castilla-La Mancha, C/Altagracia, 5013071 Ciudad Real, Spain; diegofernando.bustamante@alu.uclm.es; 3 Facultad de Arquitectura e Ingenierias, Universidad Internacional Sek, Alberto Einstein y 5ta. Transversal, Quito 170134, Ecuador; maria.mancheno@uisek.edu.ec; 4 Facultad de Ingeniería en Sistemas, Eectrónica e Industrial, Universidad Técnica de Ambato, Av. los Chásquis, Ambato 180104, Ecuador; mv.garcia@uta.edu.ec

**Keywords:** Industry 4.0, ergonomics, optimization, wearable devices, PRISMA

## Abstract

The fourth industrial revolution has transformed industrial ergonomics through the adoption of wearable technologies to enhance workplace safety and well-being. This study conducts a comprehensive scoping review, structured according to PRISMA guidelines, examining how wearable devices are revolutionizing ergonomic practices within Industry 4.0. After analyzing 1319 articles from major databases including SpringerLink, MDPI, Scopus, and IEEEXplore, 36 relevant studies were selected for detailed analysis. The review specifically focuses on how wearable technologies improve worker comfort and safety, promoting more productive work environments. The findings reveal that wearable devices have significantly impacted ergonomic conditions in industrial settings, with artificial intelligence integration showing the highest presence in analyzed applications. Over the past years, wearable technology implementations have demonstrated a 38% improvement in optimizing ergonomic conditions compared to traditional approaches.

## 1. Introduction

The integration of wearable devices and Industry 4.0 technologies in industrial settings represents a significant advancement in workplace safety and ergonomics monitoring. For the purposes of this study, wearable devices are defined as electronic or computerized devices that can be worn on the body, integrated into clothing, or implanted, capable of collecting and transmitting data about the wearer or their environment [1]. These include but are not limited to smart watches, body-mounted sensors, instrumented clothing, and biomechanical monitoring devices.

Within the context of Industry 4.0, we specifically focus on wearable technologies that incorporate one or more of the following capabilities: real-time data collection, wireless connectivity, artificial intelligence processing, or integration with broader industrial control systems. The ergonomic measures of interest in this study encompass three primary categories: (1) biomechanical factors (posture, movement, force application), (2) physiological parameters (heart rate, muscle activity, fatigue indicators), and (3) environmental interactions (exposure to vibration, repetitive motions, extreme positions) [2].

The rationale for this study emerges from three critical gaps in current research: First, while both wearable devices and Industry 4.0 technologies have been studied separately in industrial contexts, their integrated impact on ergonomic monitoring and intervention remains poorly understood [3,4]. Second, there is a lack of systematic analysis of how these technologies complement each other in practical industrial applications. Third, the effectiveness of different wearable solutions in measuring and improving specific ergonomic parameters needs comprehensive examination [5].

In industrial settings, wearable sensors have become indispensable tools for improving workplace ergonomics. These devices enable real-time monitoring of key physiological and environmental parameters, such as heart rate, body temperature, and posture. By analyzing this data, ergonomists can design customized workstations that mitigate physical and mental stress, thereby reducing injury risks and enhancing worker productivity [6]. Additionally, wearables provide immediate feedback on unsafe movements or postures, supporting continuous training and fostering safer work practices.

To address these challenges, technological advances have introduced automatic measurement and detection systems that use advanced sensors embedded in work equipment or wearable devices. These systems collect real-time data on postures, movements and physical strain, enabling ergonomists and designers to accurately identify areas of ergonomic risk and assess their impact on workers. Using advanced algorithms and artificial intelligence techniques, these systems not only collect data but also analyze patterns and trends, providing an in-depth understanding of the factors contributing to ergonomic injuries or discomfort [7].

While previous reviews have focused either on the general applications of wearable devices or their utility in specific sectors like healthcare, this study uniquely addresses the intersection of wearable sensor technologies and industrial ergonomics within the context of Industry 4.0. By leveraging PRISMA guidelines, this scoping review systematically explores the breadth of wearable device applications in enhancing workplace safety and productivity. Furthermore, it examines how emerging technologies such as artificial intelligence (AI) amplify the potential of wearables, offering insights into trends, gaps, and opportunities that previous reviews have overlooked.

The findings of this study have significant practical implications for industry stakeholders. By identifying the role of wearable devices in reducing workplace injuries, improving posture and fatigue management, and increasing operational efficiency, this study highlights pathways for cost savings through reduced absenteeism and enhanced productivity. The use of AI in wearable devices, for instance, enables predictive maintenance of workers’ health and safety, aligning with organizational goals of sustainability and workforce optimization.

This study makes several unique contributions to the field. First, it provides the most current analysis of wearable technology implementations, extending through 2024, offering insights into the latest technological developments and their applications. Second, it presents a detailed cost-benefit analysis with specific investment ranges, addressing a critical knowledge gap in the literature regarding the economic implications of wearable technology adoption. Third, it offers a novel framework for evaluating the integration of wearable devices with Industry 4.0 technologies, providing a more comprehensive understanding than previous studies. Finally, our analysis of author collaboration networks reveals emerging research patterns and centers of excellence in this rapidly evolving field.

This article is organized in five main sections, beginning with the introduction (Section 1), where the context, key definitions, and main objectives of the research are presented, along with a clear justification of the study’s rationale within the context of industrial ergonomics and Industry 4.0. Section 2 describes in detail the systematic process used to select and analyze the scientific literature, including comprehensive search strategies and explicit inclusion and exclusion criteria to ensure the relevance and quality of the studies considered.

The analytical framework is presented in Section 3, which outlines our structured approach to evaluating wearable technology implementations across multiple dimensions, including technical specifications, integration levels, and outcome measures. This section provides the foundation for our systematic analysis of how wearable technologies contribute to improved ergonomic conditions in industrial settings. The findings obtained through this analytical framework are presented in Section 4, accompanied by a critical analysis that explores their implications for both practical applications and theoretical understanding in the field of industrial ergonomics. This section synthesizes key themes and patterns identified across multiple studies, providing insights into the effectiveness of wearable technologies in various industrial contexts.

Finally, in Section 5, the key findings are synthesized and interpreted within the broader context of Industry 4.0 and industrial ergonomics. This section also presents specific recommendations for practitioners implementing wearable technologies and suggests directions for future research that could further advance our understanding of how these technologies can enhance workplace safety and efficiency.

## 2. Methodology

This research, as well as other reviews [8,9,10,11], has followed the PRISMA (Preferred Reporting Items for Systematic Reviews and Meta-Analysis) guidelines, which are designed to support authors in conducting systematic reviews, scoping reviews and other types of studies. The purpose of these guidelines is to ensure that the resulting document is unbiased and comprehensive. The first edition of the report was published in 2009, followed by an updated version in 2020 that incorporates significant advances in terms and methods. This document highlights improvements in how various studies are classified, selected, and summarized. Although originally intended to evaluate health papers, the PRISMA guidelines are now widely applied in disciplines such as education, engineering, and social sciences [12].

This guide does not seek to impose a specific format or establish a rigid sequence of steps for literature reviews. Its main objective is to ensure that all relevant information on the topic under study is adequately summarized. It is important to note that this tool should not be used to judge the methodological quality of a systematic review, although it can serve as a useful guide for critical evaluations [13].

Based on this information, this review focuses on collecting data from various prestigious databases to which the authors have access, such as SpringerLink, MDPI, Scopus, and IEEEXplore. According to [14], Scopus is the most widely used tool in academia. In addition, the review process was divided into three main stages: (i) formulation of research questions, (ii) document search and (iii) article selection.

The PRISMA framework, typically employed for systematic reviews, was adapted in this study to suit the interdisciplinary focus on wearable devices and industrial ergonomics. The review incorporates a broader range of study designs to capture the diverse applications of wearable technologies in industrial contexts. A well-defined set of inclusion and exclusion criteria was established to ensure that only relevant studies addressing wearable sensors in Industry 4.0 environments were included.

While our analysis focused on authors with multiple contributions across any author position, we acknowledge that additional relevant work exists in the field. Our selection criteria aimed to balance comprehensiveness with the need to identify established research patterns, though this may have led to the exclusion of some valuable single-publication contributions. Future reviews might benefit from exploring alternative criteria for author inclusion to capture an even broader range of perspectives in the field.

### 2.1. Research Questions

Four research questions have been posed to explore in depth the relevance of the use of 4.0 technologies in the integration of wearables in the field of ergonomics. These questions seek to delve into how advanced technologies can improve the design and implementation of wearable devices to ensure ergonomic and efficient work environments. See Table 1.

### 2.2. Document Search

The document search methodology was structured to ensure comprehensive coverage of wearable technology applications in industrial ergonomics across multiple disciplines and implementation contexts. Our systematic approach began with an extensive review of major academic databases, including SpringerLink, MDPI, Scopus, and IEEEXplore, which were selected for their comprehensive coverage of both engineering and ergonomics literature. While focusing primarily on publications from 2019 to 2024 to capture current technological capabilities and implementations, we also conducted a broader historical analysis reaching back to 2010 to understand the evolution of wearable technologies in industrial applications and establish a comprehensive foundation for our current analysis [11].

The search strategy was meticulously designed to capture the multifaceted nature of wearable technology applications in industrial ergonomics. We developed a structured search framework that incorporated multiple technological domains, application areas, and implementation contexts. This approach ensured that our search would identify not only obvious applications of wearable technology but also innovative implementations and emerging trends in industrial settings. The search terms were carefully selected based on preliminary literature review, consultation with domain experts, and analysis of keyword frequencies in relevant publications, resulting in a comprehensive set of search terms organized into three distinct categories:

- **Primary terms:** “wearable device*”, “smart wearable*”, “body-mounted sensor*”, “instrumented clothing”;
- **Context terms:** “industrial”, “manufacturing”, “workplace”, “occupational”;
- **Application terms:** “ergonom*”, “safety”, “biomechanic*”, “posture”, “movement analysis”.

The implementation of the search strategy involved a sophisticated approach to database querying and result validation. Each database was searched using carefully constructed Boolean combinations of terms from all three categories, with specific attention paid to database-specific syntax and search capabilities. The search strings were iteratively refined based on initial results, with particular attention paid to ensuring that relevant papers were not inadvertently excluded due to terminology variations. We conducted multiple pilot searches to validate our approach, analyzing the relevance and comprehensiveness of returned results before finalizing our search strategy. This rigorous validation process helped ensure that our search methodology was both sensitive enough to capture relevant studies and specific enough to maintain focus on our research objectives.

The temporal scope of our literature search was carefully considered to balance comprehensiveness with currency. While our primary analysis focuses on papers published between 2019 and 2024 to capture the most recent technological developments and applications, we recognized the importance of understanding the historical context and evolution of wearable technology in industrial settings. Therefore, we conducted a supplementary analysis of seminal papers from 2010 to 2018, particularly focusing on the following types of research:

- Foundational studies that established key principles for wearable technology in industrial applications;
- Papers that documented significant technological breakthroughs or novel applications;
- Research that established methodological frameworks still relevant today;
- Studies that provided long-term validation of wearable technology effectiveness.

The information extraction process was designed to ensure systematic and comprehensive data collection from each identified source. We developed a standardized data extraction protocol that captured not only basic bibliometric information but also detailed technical specifications, implementation contexts, and reported outcomes. This systematic approach to data collection was essential for ensuring consistency in our analysis and enabling meaningful comparisons across different studies and implementations. The extraction protocol was validated through independent review by multiple team members, with regular cross-checking to ensure consistency and accuracy in data collection. This rigorous approach to information gathering provided a solid foundation for our subsequent analysis and synthesis of findings.

- **Data extraction categories:** Study design, technology specifications, implementation context, outcomes;
- **Quality indicators:** Methodology robustness, data validity, result reliability;
- **Implementation details:** Deployment process, challenges, solutions, best practices.

### 2.3. Article’s Selection

This section was structured in three stages: Identification, Screening, and Inclusion. During the initial phase, a total of 1319 articles were identified. In the next phase, the first step was to establish the criteria for determining the eligibility of the studies. See Table 2.

**Figure 1:**
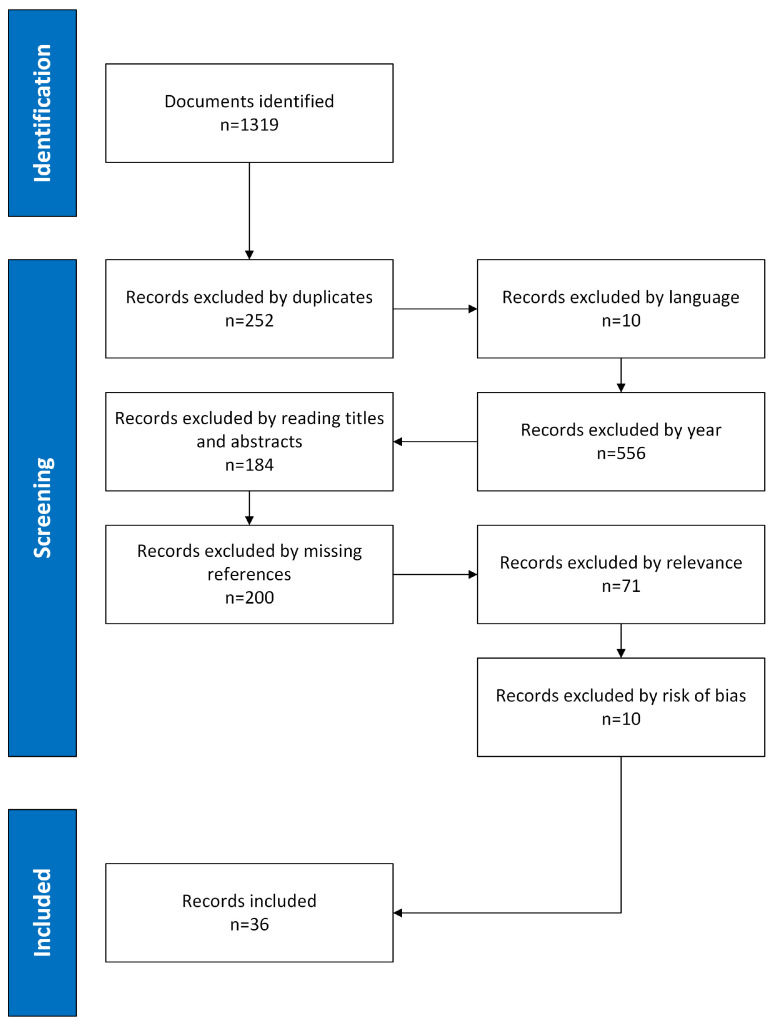
PRISMA flow diagram.

After initial sorting by relevance, title, abstract, and keywords, articles are structured in layers for further analysis. This allows prioritizing access to information on the latest available technologies. The selection process begins with the identification of documents by searching databases, resulting in 1319 initial documents. Of these, 252 were excluded as duplicates, ensuring that each document is unique for analysis. Next, 10 records were excluded because of their language, and 556 were eliminated because they did not meet the criteria of year of publication, thus maintaining the timeliness of the information. Next, a reading of titles and abstracts was performed, after which 184 articles were excluded for not having adequate information for the case study. In addition, 200 records that did not have adequate references and 71 more that did not have coherence with the topic in general were excluded. Finally, 10 articles were eliminated after assessing their bias with the Cochrane criteria. This stratified approach culminated in the inclusion of 36 full articles, which not only facilitates the identification of the most relevant and up-to-date information, but also ensures a complete and accurate assessment of each selected article. Subsequently, a thorough check was carried out to ensure that the information provided in the introduction, results, and conclusions sections is sufficient to address in depth the research questions posed in the study. See Figure 1.

**Figure 2:**
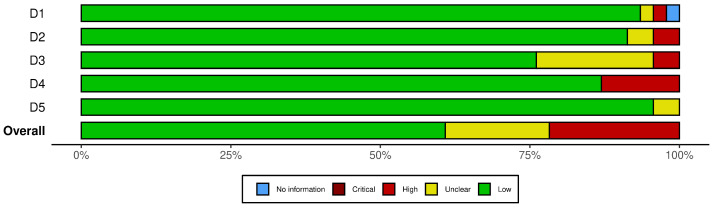
Cochrane Bias bar chart.

**Figure 3:**
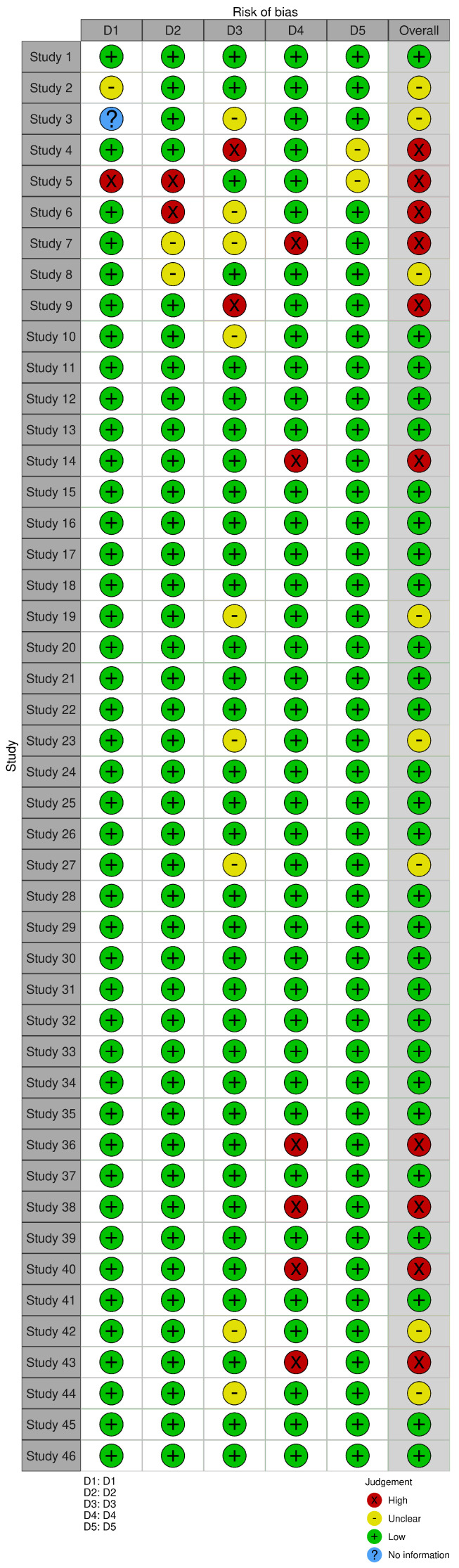
Bias analysis.

For the bias analysis in this study, the following domains were used, which were adapted from the original Cochrane bias tool:

- Inclusion criteria bias: The precise and detailed definition of selection criteria is essential to reduce significant bias in the selection of studies. In the context of Industry 4.0, this involves clearly specifying parameters such as publication period, language, quality of the source, and type of technology addressed. A well-defined criteria framework facilitates the inclusion of relevant studies and avoids the exclusion of valuable work outside the established period if its relevance is notable. This ensures that the articles selected provide valid and up-to-date information.
- Data completeness bias: Completeness and accuracy of collected data are crucial for an objective assessment of ergonomics in industrial settings. In ergonomics analysis within Industry 4.0 processes, applying rigorous methods ensures that the data captured is complete, representative, and accurate. Implementing quality controls in data collection, and verifying the consistency and completeness of the data, mitigates this bias and ensures the reliability of the results obtained.
- Methodological quality bias: Rigorously assessing the methodological quality of the included studies is fundamental. In this context, factors such as research design, sample size, blinding methods, and use of controls influence the validity of the findings. In the field of ergonomics in Industry 4.0, it is crucial to consider the quality of studies that analyze technologies such as sensors, wearable devices, and automated systems that impact the work environment. Including only studies that meet high methodological standards improves the robustness and applicability of the review.
- Bias in evidence synthesis: An objective and transparent synthesis of results is key to ensuring the credibility of conclusions. Identifying and assessing the risk of bias in the interpretation of the results helps to obtain a balanced and accurate view. In addition, the consistency and heterogeneity of the reviewed studies should be considered, as this improves the robustness of the synthesis and allows for a more accurate assessment of the effectiveness of ergonomic technologies in the context of Industry 4.0.
- Researcher experience bias: The influence of the researcher’s prior knowledge and experience on the results is an important bias to consider. In studies on ergonomics in Industry 4.0, it is critical that researchers are aware of their own biases and strive to minimize them. This ensures that the findings accurately reflect the effectiveness of the technologies evaluated in improving ergonomic conditions. In addition, transparency in documentation and article exclusion criteria allows other researchers to replicate or contrast the results obtained. The results of the 10 articles excluded in this phase can be seen in Figure 2 and Figure 3.

**Figure 4:**
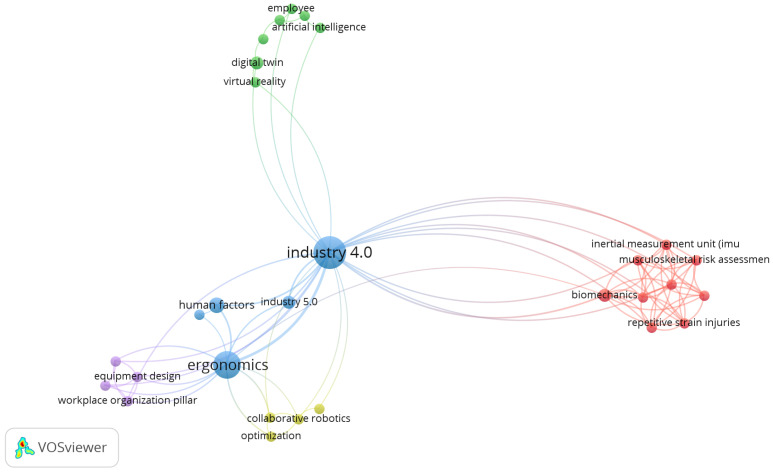
Co-occurrence analysis.

Finally, in the third phase, the 36 selected articles were reviewed again by the members of this research. In Figure 4, a network graph generated with VOSviewer representing the relationship between key concepts in the field of Industry 4.0 and ergonomics is presented. The central node of the graph is “industry 4.0”, which connects to several associated terms. On the left side, nodes such as “ergonomics”, “human factors”, and “industry 5.0” indicate a strong relationship between Industry 4.0 and ergonomics.

Concepts such as “equipment design” and “workplace organization pillar” are also connected, underlining the importance of equipment design and workplace organization in this context. Terms such as “collaborative robotics” and “optimization” are closely linked to “industry 4.0”, highlighting the relevance of collaborative robotics and optimization in this industrial revolution.

At the top left, terms such as “artificial intelligence”, “digital twin”, “virtual reality”, and “employee” show a significant connection between Industry 4.0 and advanced technologies, as well as their impact on employees. To the right of the central node, a cluster of terms related to health and biomechanics, such as “inertial measurement unit (imu)”, “musculoskeletal risk assessment,” “biomechanics”, and “repetitive strain injuries”, highlights the relationship between Industry 4.0 and musculoskeletal risk assessment and mitigation. This graphic provides a comprehensive view of how the different aspects of Industry 4.0 interrelate with ergonomics, advanced technologies and occupational risk assessment, highlighting the need for an integrated approach in the implementation of new technologies in the industrial environment.

A specific criterion has been established to determine which authors will be considered relevant for the final analysis. This criterion considers all author positions (not just first authors) in the analyzed publications. This comprehensive approach ensures we capture all significant contributors to the field, whether they are leading researchers or key collaborators.

**Figure 5:**
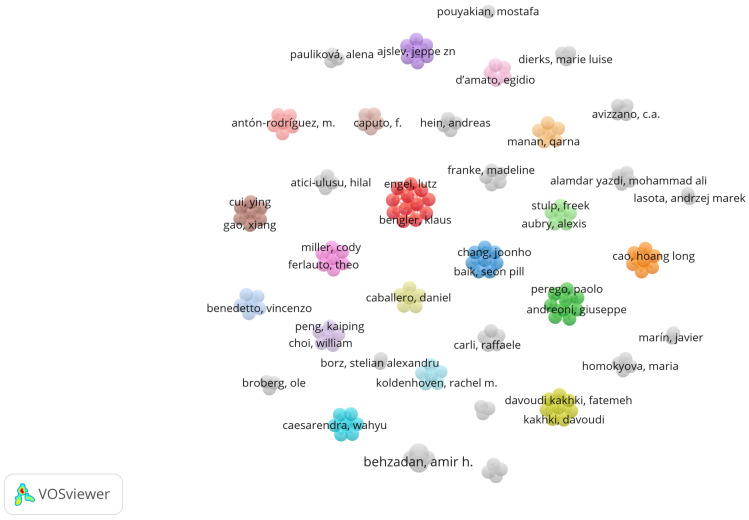
Authors analysis.

The authors analysis presented in Figure 5, as stated before, considers all author positions, not just first authors. This comprehensive analysis reveals collaboration patterns among researchers who have made multiple contributions to the field of wearable sensors in industrial ergonomics, regardless of their position in the author list. This approach provides a more complete picture of research networks and collaborative relationships in the field. Each node represents an author, while each link (edge) between nodes represents a co-authorship relationship. The size of the nodes is proportional to the number of publications an author has contributed to, while the thickness of the links represents the strength of the co-authorship ties—i.e., the more frequently two authors collaborate, the stronger their connection appears in the network. The network is divided into multiple clusters, each represented by a different color. These clusters indicate groups of authors who collaborate more frequently with each other. The visualization includes all authors in the dataset, even those who are not directly connected to the main collaboration clusters. This provides a broader view of the research landscape, including both well-integrated research groups and isolated contributors. Several dense clusters can be observed, such as the following:

- The red cluster, centered around Klaus Bengler and Lutz Engel, suggesting strong collaborative ties among these researchers.
- The green cluster, which includes Francesco Lopomo and Giuseppe Andreoni, indicating another significant collaboration network.
- The blue cluster, featuring Joonho Chang and Seon Pill Baik, highlighting another subgroup of researchers working closely together.

This visualization helps us understand the collaborative dynamics within the research community. The presence of multiple distinct clusters suggests that research on wearable sensors in industrial ergonomics is being conducted by several independent groups, rather than by a single highly connected network.

Furthermore, the variation in link strength indicates that while some authors maintain long-term and frequent collaborations, others collaborate sporadically or on a project-by-project basis. The inclusion of all authors (instead of filtering only the most connected ones) ensures that we capture the full spectrum of research contributions, from leading figures to emerging researchers in the field.

#### Articles Summary

The information found has been detailed to better visualize the data. A summary of each of the 36 final items is detailed below.

The emergence of Industry 4.0 has revolutionized manufacturing by integrating digital technologies into production processes. This paradigm shift leverages wearable technology, sensors, and augmented reality to enhance efficiency and accuracy in industrial operations. Ref. [15] **(P1)** focuses on distinguishing between correct and faulty operations in connector assembly tasks within an automotive company. To achieve this, researchers developed a digital assembly glove, a wearable prototype designed to capture vibration and force data from workers’ fingers during the assembly process.

Seventeen participants were involved in experimental trials to collect data on force and vibration signals associated with proper and defective assembly. The study employed artificial neural networks (ANNs) for signal classification, aiming to automate defect detection. The ANN model was trained, validated, and tested using the recorded measurements, ultimately achieving a 95% accuracy rate in distinguishing between correct and faulty assembly processes.

The findings underscore the critical role of wearable technology in optimizing quality control within manufacturing. By embedding real-time monitoring capabilities into production workflows, wearables such as smart gloves provide a data-driven approach to identifying errors, reducing waste, and improving overall efficiency. The study demonstrates that ANN-based analysis, when combined with wearable sensor technology, can serve as a reliable tool for defect detection, reinforcing the potential of smart production systems in modern industrial environments.

Work-related musculoskeletal disorders (WMSDs) pose a significant challenge in various industries, prompting the need for ergonomic risk assessments to regulate physically demanding tasks. Ref. [16] **(P2)** presents a methodology for automated ergonomic risk monitoring using body-mounted sensors and machine learning. By leveraging data from smartphone-based accelerometers, linear accelerometers, and gyroscopes, the system continuously tracks workers’ movements, classifies their activities, and estimates both activity duration and frequency. The methodology employs a leave-one-subject-out cross-validation framework to evaluate different data acquisition settings, including sensor placement and calibration, ensuring optimal accuracy in motion tracking.

The results demonstrate that calibrated arm-mounted smartphones can achieve a classification accuracy of 90.2%, effectively identifying task-related ergonomic risks. By post-processing the classified activity data, the system provides highly accurate ergonomic risk estimations, offering a real-time and non-intrusive approach to workplace health assessment. This research highlights the importance of wearable technology in occupational safety, demonstrating its potential to enhance timeliness and precision in ergonomic data collection and analysis. Through the integration of wearables and machine learning, this study advances workplace health monitoring, paving the way for proactive risk mitigation strategies.

Ref. [17] **(P3)** explores usability considerations in the design of glasses-type wearable computer displays, focusing on comfort and practicality. Using a structured four-step design process—preliminary analysis, idea generation, final design selection, and virtual fitting trials—researchers developed three key interventions: weight balance to reduce nasal pressure, flexible temples to accommodate different head sizes, and a hanger mechanism for seamless integration with regular eyewear. To assess effectiveness, a case study compared the newly designed 3D wearable glasses against two existing models, measuring neck muscle fatigue and subjective discomfort ratings. While there was no significant difference in muscle fatigue (*p* = 0.467), the novel design demonstrated notably lower discomfort ratings (*p* = 0.009), indicating improved user experience.

This research underscores the importance of wearable displays in industrial applications, offering insights into ergonomic improvements for next-generation smart glasses. By addressing common discomfort issues, the study provides a foundation for future innovations in wearable computing, ensuring better integration, comfort, and usability. The findings highlight how strategic design enhancements can enhance user acceptance and long-term wearability, making wearable technology more practical for diverse applications, from augmented reality interfaces to hands-free industrial computing.

The shift toward human-centered workplace design in Industry 4.0 is redefining how manufacturing environments are structured, integrating advanced methodologies such as design thinking, design doing, and design cultures. Among the most transformative technologies, Virtual Reality (VR) enables companies to validate product and process designs through immersive virtual prototypes, optimizing assembly line layouts and workplace ergonomics. This study [18] **(P4)**, introduces an innovative approach for ergonomic validation in automotive assembly lines, utilizing the ERGO-UAS system—a methodology applied by Fiat Chrysler Automobiles (FCA)—which integrates UAS for movement measurement and EAWS for biomechanical effort evaluation. By creating 3D virtual scenarios, assembly tasks can be simulated using virtual manikins, allowing for ergonomic assessment from multiple perspectives to enhance safety, efficiency, and design quality.

A key component of this methodology is the integration of wearable motion capture technology, developed by the University of Campania, consisting of inertial sensors that track human movement using sensor fusion algorithms and Kalman filtering. These wearable sensors estimate posture angles and provide data to further validate ergonomic indices such as EAWS, ensuring that workstation designs align with human capabilities and safety standards. This approach represents a significant advancement in workplace design, demonstrating how wearable technology and VR simulations can reduce costs, improve working conditions, and enhance the overall efficiency of industrial processes. The study highlights the potential of wearable motion capture to revolutionize workplace ergonomics by providing real-time, data-driven insights for optimizing human-machine interaction in manufacturing environments.

Construction work involves significant physical strain, making the reduction of workload a priority to prevent work-related injuries. This study [19] **(P5)**, explores the impact of a participatory ergonomics (PE) intervention combined with wearable technology to monitor physical workload in real-world conditions. Using surface electromyography (sEMG), inertial measurement units (IMUs), heart rate monitors, and video recordings, researchers tracked kinematic and physiological data from 80 construction workers over six months. A custom-developed program identified instances of excessive physical strain, enabling targeted ergonomic interventions. Workers were divided into a PE intervention group, which participated in structured ergonomic workshops, and a control group to evaluate differences in workload exposure.

While the intervention did not significantly reduce the number of high-exertion events, wearable sensor data revealed a notable decrease in general fatigue (*p* = 0.001) and an increase in perceived control over work tasks (*p* = 0.04) among participants. These findings highlight the potential of wearable technology in ergonomic assessments, providing real-time, objective workload measurements that enhance worker awareness and support workplace improvements. Although the intervention did not alter workload exposure, the integration of wearables offers a promising tool for long-term ergonomic strategies, helping companies optimize work conditions and mitigate occupational risks through data-driven decision-making.

This study introduces a wearable vibrotactile interface designed to alert industrial workers when adopting unfavorable postures that could lead to musculoskeletal disorders (MSDs). The system consists of up to 13 vibration motors (tactors) strategically placed across the body and integrated into motion capture workwear, serving as a real-time posture feedback mechanism. By providing haptic alerts, the system aims to improve posture awareness and reduce injury risks, particularly among older workers who are more susceptible to MSDs. To optimize notification effectiveness, the researchers evaluated pulse duration, repetition patterns, and location-based perception using data from 11 participants [20] **(P6)**.

Findings indicate that a pulse length of 150 ms, repeated two to three times, maximizes perception and ensures timely posture correction. This research underscores the importance of wearable technology in occupational safety, offering real-time, non-intrusive feedback to prevent ergonomic hazards. By seamlessly integrating with industrial workwear, the wearable vibrotactile system provides a proactive solution for posture correction, reducing strain-related injuries and enhancing workplace ergonomics. The study highlights how haptic feedback wearables can serve as an effective intervention, promoting safer working conditions through immediate, data-driven feedback mechanisms.

Ref. [21] **(P7)** presents a wearable motion capture (MoCap) system designed to track and assess worker postures in industrial environments, aligning with the human-centered approach of Industry 4.0. The system, composed of inertial measurement units (IMUs), estimates the orientation of key body segments using Kalman filtering to evaluate posture angles during movement. Its modular design, consisting of four sensor modules, enables real-time ergonomic analysis, allowing companies to optimize workplace design based on data-driven insights. By integrating wearable technology, this system provides continuous posture tracking, facilitating proactive interventions to prevent ergonomic risks in assembly activities.

The wearable MoCap system generates motion data that can be used to develop automated algorithms for ergonomic assessment, ensuring real-time evaluation of posture scores. The test case conducted in an industrial setting demonstrated the system’s reliability, highlighting its potential for enhancing workplace ergonomics and reducing strain-related injuries. This research reinforces the importance of wearables in industrial process design, offering a scalable and efficient solution for monitoring human motion and improving ergonomic conditions. By leveraging wearable sensor technology, industries can make informed decisions to enhance worker safety, comfort, and productivity.

The feasibility of using wearable technology to detect driver drowsiness, a major cause of accidents in the transportation industry, is described by [22] **(P8)**. Researchers developed a Google Glass-based detection system that utilizes its proximity sensor to monitor eye blink frequency, a key indicator of fatigue. A simulated driving study was conducted to validate the system, comparing driving performance and blink patterns between alert and drowsy states. The results showed that drowsy drivers exhibited increased blink frequency, slower braking response times, and greater lane deviation, confirming the correlation between drowsiness and impaired driving performance.

By implementing a threshold-based algorithm, the wearable proximity sensor proved to be a reliable tool for detecting eye blinks and distinguishing between alert and fatigued states. This research highlights the potential of wearable devices in real-time driver monitoring, offering a non-intrusive, continuous, and automated solution for drowsiness detection. The findings demonstrate how wearable sensor technology can enhance transportation safety, with applications not only in driving but also in aviation and other fields where operator alertness is critical. By integrating wearables into fatigue detection systems, industries can reduce drowsiness-related incidents and improve overall safety.

The use of wearable sensors to monitor and model physical fatigue in the workplace, an issue that impacts both productivity and worker safety, has been extensively discussed in the past few years [23] **(P9)**. While wearables have been widely used to track fatigue in athletics, transportation, and mining, their application in manufacturing ergonomics remains underdeveloped. To address this, researchers examined how wearable sensor data could detect the onset of fatigue during simulated manufacturing tasks and estimate fatigue levels over time. Sensory data from five different sensor placements were collected from eight participants, and penalized logistic regression was used for fatigue detection, while multiple linear regression estimated fatigue progression.

By applying Least Absolute Shrinkage and Selection Operator (LASSO) for feature selection, the study demonstrated that wearable sensor data can accurately predict fatigue states and provide insights into its development over time. The proposed data-driven approach is adaptable across different workload conditions and industrial settings, making it a scalable solution for fatigue management. This research reinforces the importance of wearable technology in workplace safety, offering real-time, objective fatigue assessment that can be integrated into ergonomic interventions. By leveraging wearable sensors for fatigue monitoring, industries can develop preventive strategies to reduce workplace injuries and optimize worker performance.

In order to create a complete solution that encompassed both physical and technological aspects, with the aim of providing an affordable and practical option for assessing ergonomics in the automotive industry, the researchers devised and created a system that combines affordable sensors with easily accessible technologies. With this system, it is possible to record real-time data on employees’ posture and movements as they perform their work tasks. In ref. [24] **(P10)**, inexpensive motion sensors and cameras were used to collect accurate data about the positions and movements of all body parts. The data were then subjected to ergonomic analysis algorithms that examine the potential for injury and suggest changes to improve workstation layout.

Both the hardware for data capture and the software required for processing and analysis are included in the pipeline. An app was created that enables the visualization and analysis of the collected data, providing detailed reports along with specific recommendations. The purpose of this system is to provide a comprehensive and easily applicable solution for the automotive industry, in order to increase both the safety and well-being of employees without generating excessive costs. The study showed that the system is reliable and effective in detecting ergonomic hazards, allowing companies to take preventive action and optimize working conditions in their facilities.

This work [25] **(P11)**, deploys the use of wearable mobile sensors to assess and improve ergonomic conditions in construction work, a labor-intensive industry where sustained physical exertion can lead to musculoskeletal disorders (WMSDs). Traditional ergonomic risk assessments are often time-consuming and impractical for dynamic construction sites, making wearable technology an essential tool for real-time posture monitoring. This research introduces a low-cost, smartphone-based system that utilizes built-in sensors to track workers’ trunk and shoulder flexion, identifying potential ergonomic hazards without the need for manual observation. By leveraging wearable mobile sensing, the system provides an autonomous, unobtrusive method for detecting unsafe postures and assessing physical strain.

The findings reveal that smartphone sensor measurements closely align with traditional observational assessments, demonstrating their reliability in ergonomic analysis. This wearable-based approach is not only applicable to construction workers but also to other physically demanding professions such as carpenters, welders, farmers, and healthcare assistants. By integrating wearable technology into occupational safety protocols, industries can proactively monitor, evaluate, and mitigate ergonomic risks, ultimately reducing workplace injuries and improving worker well-being. This study highlights how ubiquitous, wearable sensing solutions can revolutionize ergonomic assessments, making them more scalable, cost-effective, and adaptable to diverse work environments.

The development of a co-simulation environment that employs digital twins and virtual reality for the purpose of designing and evaluating industrial workstations is being developed at [13] **(P12)**; designers and evaluators have the ability to interact with accurate virtual representations of workstations. This makes it easier to detect ergonomic and efficiency issues before they are physically built. Real data from existing workstations was used to generate the digital twins, ensuring maximum accuracy and realism in the virtual simulations.

In the evaluation, work tasks are simulated in a virtual environment so that evaluators can observe and analyze how workers would interact with workstations. Users have the ability to test different configurations and settings in real time, allowing them to optimize the ergonomic design and improve their operational efficiency. It was demonstrated through the results that the joint use of digital twins and virtual reality represents an effective tool to anticipate possible inconveniences and make corrections before carrying out their physical implementation, which translates into a lower economic and temporal expense. Additionally, it makes it easier for operators to actively intervene in the design process to ensure the practicality and user-focused nature of the proposed solutions. In conclusion, the study showed that the combination of digital twins and virtual reality in a co-simulation provides a novel and effective way to improve both the design and the evaluation of industrial stations. This is especially beneficial for achieving greater ergonomics and efficiency in such spaces.

The authors conducted a thorough investigation of the available literature in order to identify trends, challenges, and possibilities regarding the combination of advanced technologies and ergonomic practices in industrial settings. The article [26] **(P13)** examined how rising technologies, such as artificial intelligence, robots, the Internet of Things (IoT) and cyber–physical systems, are changing workplaces and introducing new ergonomic and human factors concerns.

The effectiveness of a passive back support exoskeleton (BExo) in reducing perceived physical exertion and improving ergonomic safety in the manufacturing industry was developed in the study [27] **(P14)**. Twenty-two university students performed manual material handling tasks in a controlled environment, with and without the BExo, and completed a questionnaire about their experience. ANOVA was used to analyze biomechanical strain on various body parts.

The results showed that the BExo significantly reduced discomfort and physical strain in the lower back, shoulders and knees, improving ergonomic posture and reducing fatigue. These findings highlight the potential of passive exoskeletons to improve worker safety and efficiency in industrial settings. In order to conduct their research, the authors compiled and evaluated previous studies addressing different aspects of Industry 4.0 and how it affects ergonomics and human factors. Key areas of interest were identified including human–robot collaboration, human-machine interface design, cognitive load of workers, and technological adaptation to customize workstations. Also, the authors addressed the potential impact of these technological advances on employee health, safety, and well-being.

The article [28] **(P15)** presents a methodological framework that explores the use of digital human modeling (DHM) to improve ergonomics in industrial environments. The main objective is to employ simulation and digital modeling technologies to analyze, predict, and optimize working conditions, with a focus on reducing ergonomic risks and improving workers’ well-being. The article describes how DHM makes it possible to simulate work tasks and assess the physical impact on workers without the need for lengthy physical testing, which streamlines the process of designing and adjusting workstations. It also highlights technological tools such as 3D modeling software and sensors that capture human movements for accurate and detailed analysis. In the methodological framework, the article proposes key steps that include the collection of data on postures, movements, and loads faced by the worker, as well as the creation of virtual models that simulate such activities. These models allow work environment designers and ergonomists to predict physical wear and tear and make adjustments before implementing changes in the real space.

A new way to address current needs in different work environments was proposed using motion capture as a basis. The focus of the research is the development and application of a motion capture system that provides a thorough real-time analysis of workers’ postures and movements [29] **(P16)**. Advanced sensor technology and biomechanical analysis algorithms will be used; this method allows an accurate and dynamic assessment of ergonomic risks. This facilitates the rapid detection of problem areas and the possibility of implementing improvements in the design of both tasks and the work environment.

The results of the study revealed that the methodology focused on movement monitoring not only increases the accuracy of ergonomic assessments, but also enables a more agile and effective intervention to correct posture and movement problems. According to the authors, this technology is especially effective in detecting risk factors that are not easily identified by traditional ergonomic assessment methods. Also, this methodology achieved a considerable decrease in the number of workplace injuries and improved both the efficiency and overall well-being of employees. In conclusion, the study indicates that incorporating motion capture into ergonomic assessments is an important step forward in promoting safer and healthier work environments.

This study, [30] **(P17)**, examines the impact of offshore shiftwork on worker fatigue using wearable physiological sensors to monitor heart rate variability (HRV). Workers in oil and gas extraction (OGE) face long shifts, physically and mentally demanding tasks, and irregular schedules, which contribute to fatigue-related safety risks. To assess these effects, wearable HRV monitors were used to track ten offshore operators over six consecutive workdays on a drillship. HRV was analyzed in frequency (LF/HF ratio) and temporal (RMSSD) domains, with results showing that night shift workers exhibited greater fatigue levels, and swing shifts exacerbated physiological strain.

The findings highlight the importance of wearable technology in objectively assessing workload and fatigue levels in high-risk environments. By continuously monitoring HRV responses, wearable sensors provide real-time insights into the physiological impact of shift schedules, enabling companies to develop data-driven fatigue management strategies. The study underscores the potential of wearables in occupational health, offering a proactive approach to mitigating fatigue-related risks in offshore industries and improving worker safety through personalized, physiological-based monitoring systems.

The impact of implementing ergonomic principles to reduce both the physical burden on employees and operating costs was examined in one company. The research was carried out in an industrial environment, in which different ergonomic interventions were applied to determine their influence on both the well-being of workers and the overall performance of the company. The authors of the study [31] **(P18)** analyzed the work areas that present a high ergonomic risk and suggested practical and cost-effective solutions for the company.

This article reveals that by implementing ergonomic improvements, there is a marked decrease in the occurrence of musculoskeletal disorders among workers, which in turn reduces absenteeism and boosts productivity. Likewise, there is a considerable decrease in expenses related to work-related injuries and time off work. Not only do ergonomic interventions such as optimizing workstations and adding auxiliary mechanical equipment also generate a positive return on investment for the company. To summarize, the study provides evidence that ergonomics is beneficial for both employee health and performance, as well as for reducing costs and improving business competitiveness.

A novel wearable system for real-time assessment of biomechanical load in repetitive tasks is introduced by [32] **(P19)**. Unlike traditional assessment methods that rely on subjective visual inspection and manual evaluations, this wearable wireless system provides continuous, objective monitoring of muscular efforts and postures directly in the workplace. Using inertial measurement units (IMUs) and surface electromyography (sEMG) sensors, the system accurately tracks upper limb movements and assesses forearm muscle strain. By applying Rapid Upper Limb Assessment (RULA) and Strain Index (SI) methodologies, it offers a comprehensive, automated evaluation of the risks associated with repetitive efforts, facilitating timely interventions to prevent WMSDs.

The system’s non-obtrusive design enables workers to perform their daily tasks without interruption, making it suitable for monitoring in real-world industrial environments. Initial tests conducted with supermarket cashiers demonstrated its capability to detect ergonomic risks effectively, showcasing its potential to reduce WMSD-related injuries and associated costs. This research highlights the critical role of wearable technology in occupational health, providing a data-driven, real-time approach to identify and mitigate ergonomic risks, ultimately enhancing workplace safety and efficiency. By integrating wearable sensors into routine operations, industries can proactively address WMSD factors, improving worker health and reducing downtime due to injury.

A novel approach to ergonomic risk assessment focused on creating an automated system that can constantly monitor and evaluate employee postures and movements at all times. The study, [33] **(P20)**, uses computer vision techniques and advanced machine learning algorithms to capture and analyze workers’ posture, with the goal of identifying situations that may present ergonomic risks. Additionally, collaborative robots are part of the process to assist workers in specific tasks, decreasing the physical burden and optimizing work efficiency.

The study findings suggest that the proposed system proves to be highly effective in detecting and reducing ergonomic hazards in the work environment. The 3D human pose assessment enables the detection of behaviors that could cause musculoskeletal injuries, providing an accurate and detailed assessment of postures. It not only relieves the physical burden on workers, but also improves performance when performing repetitive or heavy tasks collaborating with robots. The system, as a whole, has the potential to generate a safer and healthier work environment, which not only boosts productivity, but also decreases costs resulting from workplace accidents. The authors conclude that incorporating advanced technologies in ergonomic assessment is an important advance in improving working conditions in different industrial sectors.

Study [34] **(P21)** was commissioned to analyze ergonomic disparities between the effects of surgeons’ postures during surgical procedures. The study investigators analyzed a series of surgical procedures performed both conventionally and using advanced sensors and motion capture technology. Records were taken of surgeons’ posture, physical exertion, and comfort during surgery to contrast the ergonomic hazards associated with each procedure.

The study revealed that robotic-assisted surgery has significant ergonomic benefits compared to conventional laparoscopy. At the end of the procedures, surgeons using robotic systems reported experiencing less awkward postures and decreased physical fatigue. The motion capture data corroborated that robots enable superior precision and control of movements, decreasing physical stress on surgeons. Also, the use of assistive robots helped improve overall ergonomics by reducing the risk of musculoskeletal disorders. The study indicates that the use of robotic technology in laparoscopic surgery not only optimizes surgical procedures and outcomes, but also promotes a safer and healthier working environment for physicians.

The authors of the article [35] **(P22)** carried out an experimental study where they created a system based on acceleration sensors. This system was intended to monitor and record participants’ back movements and postures during different work activities. The main purpose was to use this information to identify and accurately categorize the different forms of physical activity that have an impact on the back, which will help to detect possible ergonomic risks at an early stage.

The results of the study showed that the system using accelerometers can effectively distinguish between physical and static back activity, as well as different positions. Thanks to the data collected, the researchers were able to identify specific movement and loading patterns that are linked to an increased risk of musculoskeletal injuries. Valuable information on how to improve ergonomic conditions in the workplace through adjustments to workers’ tasks and postures was also obtained through analysis of the acceleration data. To summarize, Muşat and Borz point out that acceleration sensors are a promising option for assessing ergonomics at work, as they allow for accurate and effective monitoring of workers’ health and well-being.

The article [36] **(P23)** analyzes how optimization using the Particle Social Optimization (PSO) algorithm can be used to improve ergonomic posture during human–robot interaction in a virtual environment. The research was conducted in a simulated environment, where interactions between humans and robots working together were evaluated. The researchers used the PSO algorithm with the aim of optimizing workers’ postures, which reduced ergonomic risks and improved both the level of comfort and efficiency during task execution.

It was proven through the study that using PSO in posture optimization is an effective way to improve ergonomics in human–robot collaborations. Through the use of the PSO algorithm, optimal postural configurations were found that resulted in a significant decrease in physical loads and awkward postures. Additionally, the use of simulation in a virtual environment gave the researchers the possibility to test and modify different task configurations without putting the participants at risk. This allowed for safe and efficient implementation in real environments. In summary, the study points out that using particle swarm optimization may be a promising way to improve ergonomics in human–robot collaborations. This has important benefits for both health and work efficiency.

The impact on occupational health and safety, as well as quality requirements, of implementing collaboration between humans and collaborative robots (cobots) in manufacturing is being investigated. The study [37] **(P24)** conducted empirical research in a manufacturing environment, examining quantitative and qualitative information collected through surveys, direct observations, and analysis of safety and quality records. Statistical techniques were used to assess how human–cobot collaboration affects the incidence of workplace accidents, workload, staff stress level, and final product quality. The implementation was carried out in different phases, allowing a comparative evaluation before and after the incorporation of the cobots.

According to the results of the study, it could be observed that the collaboration between humans and cobots generates a beneficial influence in terms of occupational health and safety, achieving a considerable reduction in both occupational accidents and the physical burden imposed on employees. In addition, a reduction in work stress could be noted thanks to the assignment of repetitive and physically demanding tasks to the cobots. The research demonstrated that the incorporation of cobots contributes to meeting demanding quality standards by improving accuracy and consistency in the production of manufactured products. However, the authors also emphasized the importance of providing adequate and consistent training to employees, as well as adapting both work procedures and organizational culture to optimize the advantages offered by this technology.

In [38] **(P25)**, a new word was developed to describe ergonomics when applied to Industry 4.0 technologies. The author suggests the word “cyberergonomics” as a concept that encompasses the merging of traditional ergonomics principles with advanced technologies such as artificial intelligence, the Internet of Things (IoT), and robotics. Pouyakian supports this proposition through a thorough analysis of how ergonomics has evolved and the ever-increasing technological demands in contemporary workplaces. To identify gaps in current terminology and their impact on the effective implementation of ergonomic solutions in contemporary industry, a comprehensive literature review can be employed along with detailed case study analysis.

According to his findings, using the term “cybergonomics” can contribute to a better global and accurate understanding of the ergonomic challenges related to Industry 4.0 technologies. According to the author, this new terminology facilitates the inclusion of ergonomic aspects in the design and application of advanced technological systems, thus promoting well-being and occupational health. In addition, Pouyakian highlights that using this term could encourage exploration and progress in the field of ergonomics, encouraging experts to approach emerging ergonomics-related challenges with novel and adjustable ideas. In summary, the “cybergonomics” approach provides a solid conceptual basis for addressing the ergonomic challenges of the future and enhancing the quality of work life in highly technological environments.

In [39] **(P26)**, a path planning method for collaborative robots that meets the requirements of safety, ergonomics, and time efficiency is developed. The researchers created an algorithm that combines safety and ergonomics models for the purpose of generating time-efficient paths while reducing risks to human workers. They validated their approach by using advanced mathematical optimization techniques and computational simulations. A study was conducted that consisted of creating an experimental environment to test different interactions between robots and humans. During this experiment, the performance of the algorithm was evaluated in terms of safety compliance, ergonomic load reduction, and time efficiency.

According to the study, the algorithm developed by Putri and his team was found to be successful in creating safe, ergonomic, and time-efficient routes for collaborative robots. Human operators experienced a significant reduction in risk exposure and an improvement in the ergonomics of the work environment, which led to the prevention of injuries and improved worker well-being, as demonstrated by the simulations. Also, thanks to the algorithm, it was possible to optimize the robots’ operating time, resulting in increased productivity and efficiency in collaborative manufacturing procedures. According to the authors, their method represents an important progress towards the successful incorporation of collaborative robotics in the industrial sector by offering a balanced solution that meets the demands in terms of safety, ergonomics, and operational efficiency.

The article [40] **(P27)** proposes the creation of a method to plan trajectories for collaborative robots, focusing on meeting the highest standards of safety, ergonomics, and time optimization. The authors have created a revolutionary algorithm that combines safety and ergonomics when designing paths, taking advantage of advanced mathematical optimization techniques. In order to verify their approach, they conducted computer simulations and set up an experimental environment in which they tested various robot–human interaction scenarios. The focus of the study was to evaluate how the algorithm would reduce risks to human operators, improve ergonomic conditions, and maximize the efficiency of robot operation time.

According to the results of the study, it is concluded that the algorithm proposed by Putri and colleagues is highly effective in generating trajectories for collaborative robots. Both simulations and experiments showed that the method considerably decreases the risks related to human–robot interaction, which increases safety and reduces the physical effort for operators. The algorithm, in addition to enabling the optimization of operation times, succeeded in increasing both efficiency and productivity in collaborative manufacturing processes. According to the authors, their approach represents a major breakthrough in achieving safe and efficient integration of collaborative robots in industrial environments by simultaneously addressing the aspects of safety, ergonomics, and time performance. This offers a balanced solution, they say.

The use of artificial neural networks (ANN) for the classification of human gait patterns with a view to optimizing the ergonomic development of lower limb prostheses has become popular in recent years. In [41] **(P28)**, gait analysis data were recorded by the authors, who used motion sensors and force platforms to collect different metrics related to how the participants in the group walked. These data were then used to train a model of ANNs with the ability to detect and categorize various movement patterns. To ensure the accuracy and robustness of the system, cross-validation techniques were used to validate the model. In addition, prior to this, data preprocessing and careful selection of relevant features were carried out using a comprehensive methodological approach.

Putri and colleagues have been able to demonstrate, through the results obtained in the study, that the ANN model can classify different human gait patterns with high accuracy. The accurate classification capability is essential for designing and fitting lower limb prostheses in a customized manner, which ensures that they are correctly adapted to the individual needs of users. Also, the study emphasized that the implementation of ANN in prosthesis ergonomics results in both functional improvements and improvements in comfort and fatigue reduction for wearers. To summarize, according to the research, the use of advanced artificial intelligence techniques such as ANNs can result in considerable improvements in the quality of life of those who rely on both the study of walking and prosthetic design and use.

The development of wearable technology for spaceflight applications, focusing on the Electronic-Textile System for the Evaluation of Wearable Technology (E-SEWT), designed at NASA’s Wearable Electronics Application and Research Lab (WEAR Lab), is proposed by [42] **(P29)**. The E-SEWT project aims to enhance astronaut performance aboard the International Space Station (ISS) by integrating electronic sensing, computing, and interaction capabilities into a comfortable, form-fitting garment. Unlike conventional wearables, this system consists of a base unit with modular sensor swatches, allowing astronauts to customize their smart garments based on specific tasks or personal preferences. This reconfigurable design not only improves ergonomics and safety but also facilitates easy hardware updates and replacements, ensuring adaptability in space environments.

By prioritizing mobility, comfort, and data efficiency, the project refines wearable garment design through continuous user interaction and testing. The modular nature of the system allows for task-specific sensor integration, optimizing data collection and usability in space operations. This research highlights the importance of wearable e-textiles in augmenting human capabilities, improving efficiency and autonomy in space missions, and setting a foundation for future wearable applications in extreme environments. The E-SEWT project demonstrates how wearable sensor systems can revolutionize human–machine interaction in aerospace settings, paving the way for more adaptive, multifunctional smart garments in both space and terrestrial applications.

The construction of a versatile and adaptable laboratory that aims to investigate the interaction between humans, robots, and neuroergonomics was created. The team in charge of this project created an experimental setup that enables flexibility and adaptability in different research on human–robot interaction as well as cognitive ergonomics. The laboratory has hardware and software modules that can be easily readjusted to adapt to different experimental situations. In [43] **(P30)**, high-tech devices were used to monitor the physiological and neurological reactions of the subjects, such as EEG, eye tracking and advanced motion sensors. The experimental environment was also designed to simulate realistic human–robot collaborative work situations.

The effectiveness and versatility of the modular lab was demonstrated in a wide range of neuroergonomic and human–robot interaction studies. Initial experiments conducted in this environment provided accurate data on the cognitive and physical responses of participants while interacting with collaborative robots. The results indicated that the lab setup allows for easier detection of key ergonomic and neurocognitive factors that influence efficiency and safety during human–robot interaction. Additionally, the information collected has contributed to the development of more effective interface designs and interaction protocols that improve collaboration and decrease the cognitive stress experienced by users. To summarize, the development of this versatile and flexible laboratory is an important advance in neuroergonomics and human–robot interaction research. This innovative environment provides a solid and adjustable foundation for future research in this area.

The article [44] **(P31)** integrated digital ergonomics and digital work planning in university education in Germany and Austria. Collecting data from various sources, such as student and faculty surveys, in-depth interviews with educators, as well as content analysis of syllabi and course materials, the authors conducted their research. In addition, direct observations of classes and workshops were conducted to assess the implementation and use of digital ergonomics tools and concepts in an educational setting. The focus of the study was to identify educational practices that work and obstacles related to teaching these topics at a university.

Incorporating digital ergonomics and digital work planning into university educational programs was found to have a positive impact on preparing students for the digitized world of work, according to study results. Students who engaged in these programs exhibited a deeper understanding of ergonomic principles applied to digital technologies and gained practical skills for using digital tools in work planning. In addition, the authors noted several obstacles that must be addressed, such as the need to keep course content up to date with ongoing technological advances and to find an appropriate balance between theory and practice during the training process. The overall conclusion of the study is that university education should include the integration of digital ergonomics and digital work planning, as this is essential to prepare future professionals with the skills required to meet the challenges arising from digitization in the work environment.

The study [45] **(P32)** focused on improving health literacy among individuals with chronic diseases. The research focused on evaluating and implementing interventions that promote health literacy, addressing both the benefits and challenges at the personal and community levels. It emphasized the importance of local community participation and collaboration in designing effective interventions, especially in resource-limited settings.

The findings indicated that interventions designed in collaboration with the community were more successful and sustainable, highlighting the relevance of local ownership of the proposed actions. In addition, the importance of electronic health literacy (eHealth), which can be learned and is crucial for improving health outcomes in people with chronic diseases, especially in contexts where online information can be overwhelming, was highlighted. In conclusion, the study emphasizes the need for specific online programs to promote physical activity and reduce stress, which can significantly contribute to improving health-related quality of life in this vulnerable population.

In recent years, the use of Extended Reality (XR) technologies has been explored to improve the performance of workers in industrial assembly operations. In [46] **(P33)**, a flexible XR solution was developed to assist workers in the assembly of medium-voltage switchgear. The method consisted of integrating augmented reality (AR) and virtual reality (VR) technologies to provide real-time instructions and reduce cognitive load. Workers used assembly manuals adapted to their skills, thus improving the accuracy and efficiency of the process.

Results showed that the implementation of XR significantly reduced training time, decreased assembly errors, and increased overall productivity. In addition, workers experienced increased job satisfaction due to the intuitive interfaces and supportive environment created by XR technologies. In summary, the study demonstrated that XR technologies can significantly improve workers’ cognition and performance in industrial assembly operations.

A systematic review to identify portable devices used for ergonomic purposes in the scientific literature was developed by [47] **(P34)**. Twenty-eight articles were retrieved and analyzed using eleven dimensions of comparison related to ergonomic factors, purposes and criteria, populations, application, and validation. Devices reviewed included sensor systems composed of different types and numbers of sensors located on various parts of the body. Also reviewed were smart watches, body-mounted smart phones, insole pressure systems, and vibrotactile feedback interfaces, all used to assess and/or monitor physical loads or postures.

The results of the review revealed that most of the available devices are sensor systems, which represent the most widely used technology for monitoring and reducing the risk of awkward postures. Wearable devices, such as smart watches and insole pressure systems, have been shown to be effective in assessing and monitoring users’ physical loads or postures. The analysis framework defined in the study provides an overview of the state of the art of smart wearables in ergonomics and supports the selection of the most suitable devices for industrial and non-industrial settings. In addition, the results suggest future research directions in improving ergonomic conditions through the use of wearable technology.

The objective of improving walking endurance and reducing metabolic consumption of the lower limbs, using an ergonomic design based on the biological structures of the lower limbs by constructing an auxiliary force profile that mimicked the biological strength of the Achilles tendon, was developed by [48] **(P35)**. To replicate this auxiliary profile, an iterative learning control was applied that iteratively modified the traction displacements of the motor units. The exoskeleton was evaluated by performance experiments involving four subjects walking on a treadmill at different speeds and inclines.

The results showed that wearing the A-Suit significantly reduced the mean heart rate, an index of metabolic consumption, compared to walking without the suit. At a moderate speed of 1.25 m/s, the mean heart rate in the condition with the suit on was 7.25 ± 1.32 and 14.40 ± 2.63 lower than in the condition without the suit. However, the additional mass of the A-Suit led to a maximum increase in heart rate of 7.83 ± 1.44. Overall, the reduction in heart rate with the suit on at different slopes was 6.93 ± 1.84 and 13.4 ± 1.93 compared to the no-suit condition. These results indicate that the A-Suit is effective in reducing energy consumption during walking, suggesting its feasibility for improving human assistance in medical applications and other purposes.

In [49] **(P36)**, a system was created to manage parking availability using sensor data and predictive algorithms. The process included designing the system architecture, implementing sensors, and developing algorithms to predict parking space availability. The results showed that the system effectively managed parking spaces, reducing the time spent searching for parking and improving overall efficiency.

## 3. Analysis Framework

The analytical approach employed in this study was designed to provide a comprehensive understanding of how wearable technologies are transforming industrial ergonomics practices. We developed a multi-layered framework that examines not only the technical aspects of wearable implementations but also their practical impact on workplace safety and efficiency. This systematic approach enabled us to identify patterns, trends, and best practices across diverse industrial settings, while maintaining focus on the specific contributions of wearable technologies to ergonomic improvements. Through careful consideration of both quantitative and qualitative factors, we ensured that our analysis captured the full spectrum of benefits and challenges associated with wearable technology adoption in industrial environments.

The integration of wearable devices within Industry 4.0 frameworks represents a complex interplay of technical, human, and organizational factors. Our analysis framework was specifically designed to capture these multifaceted relationships through a structured evaluation approach that considers both direct and indirect impacts of wearable technology implementations. By examining how different elements of wearable solutions interact with existing industrial systems and processes, we were able to develop a more nuanced understanding of their effectiveness in real-world applications. This comprehensive perspective was essential for identifying not just successful implementations, but also understanding the underlying factors that contribute to their success.

The methodological rigor of our analysis was ensured through the development and validation of standardized assessment tools and protocols. We established clear criteria for evaluating the quality and reliability of reported results, with particular attention paid to the verification of measurement accuracy and the validation of implementation methodologies. This systematic approach to quality assessment helped ensure that our findings were based on robust evidence and reliable data, providing a solid foundation for practical recommendations and future research directions. The framework was iteratively refined based on initial analysis results to ensure comprehensive coverage of all relevant aspects of wearable technology implementation.

The reviewed studies were analyzed using a structured framework focusing on four key dimensions:

- **Technology Implementation:** Type of wearable device, sensors used, data collection methods;
- **Integration Level:** Standalone operation vs. integration with other Industry 4.0 systems;
- **Ergonomic Parameters:** Specific measures monitored and assessed;
- **Outcome Measures:** Quantitative and qualitative results reported.

To facilitate systematic analysis across multiple studies and implementation contexts, we developed a comprehensive data synthesis approach that enabled meaningful comparisons while accounting for variations in study design and reporting methods. This methodology allowed us to identify common patterns and trends across different industrial sectors and application types, providing insights that would not be apparent from individual case studies alone. The synthesis process was carefully structured to maintain objectivity while ensuring that important contextual factors were appropriately considered in our analysis.

Our analytical framework incorporated both quantitative metrics and qualitative assessments to provide a holistic understanding of wearable technology effectiveness. The quantitative analysis focused on measurable outcomes such as injury reduction rates, productivity improvements, and system reliability metrics, while qualitative assessments examined factors such as user acceptance, implementation challenges, and organizational impact. This dual approach enabled us to develop a more complete picture of how wearable technologies contribute to improved ergonomic conditions in industrial settings.

The synthesis of findings across multiple studies required careful attention to methodological consistency and result comparability. We developed standardized protocols for data extraction and analysis, ensuring that information from different studies could be meaningfully compared and integrated into our overall findings. This systematic approach to data synthesis helped identify not only common trends but also unique insights that emerged from specific implementation contexts.

For data extraction and synthesis, we developed a standardized form capturing the following information:

- Study characteristics (design, sample size, duration);
- Technology specifications and capabilities;
- Implementation context and challenges;
- Reported outcomes and effectiveness measures;
- Integration with existing industrial systems.

Rather than presenting individual study summaries, we synthesized findings according to key themes and patterns identified across multiple studies. This approach provides a more meaningful analysis of the collective evidence regarding wearable technology effectiveness in industrial ergonomics.

## 4. Results and Discussion

### 4.1. Critical Information Extracted from the Selected Papers

Table 3 presents a selection of relevant articles, systematically detailing the year of publication, the Industry 4.0 technologies implemented in each study, and a concise but technical description of their application and usefulness in the field of industrial ergonomics. The summaries provided include the main objectives of each research, the context of implementation of the emerging technologies, and the tangible and intangible benefits identified in terms of ergonomics, productivity, and occupational safety.

Each table entry highlights how advanced technologies, such as cyber–physical systems, artificial intelligence, augmented reality, and human–robot interaction, are revolutionizing ergonomic practices. For example, included are studies demonstrating the use of real-time monitoring systems to assess occupational fatigue, the ergonomic design of automated and adaptive workstations, and the use of multi-objective methods to simultaneously optimize occupational well-being and economic efficiency.

In addition, research focused on the digital transformation of small and medium-sized enterprises (SMEs) is addressed, highlighting the integration of Internet of Things (IoT) technologies and their impact on the creation of emerging sub-disciplines within industrial ergonomics. Other studies emphasize safe collaboration between workers and robots, the design of workspaces optimized through virtual and augmented reality, and the development of collaborative assemblies that prioritize both ergonomics and economics.

Taken together, the table provides a comprehensive and technically rigorous overview of current applications of 4.0 technologies in improving ergonomic conditions in industry. This compilation not only evidences significant advances in occupational safety and comfort, but also underscores the ability of these technologies to profoundly transform work environments toward greater sustainability, efficiency, and worker well-being.

#### 4.1.1. How Specific Technologies Outperform Others in Ergonomic Optimization

Artificial intelligence (AI) and virtual reality (VR) have demonstrated transformative potential in optimizing ergonomic conditions, surpassing traditional methods in accuracy, adaptability, and impact. These technologies leverage advanced computational capabilities and immersive environments to address ergonomic challenges dynamically and effectively.

AI excels in processing large datasets generated by wearable sensors, enabling detailed analysis of complex patterns associated with ergonomic risks, such as repetitive strain injuries, improper posture, and prolonged fatigue. By employing machine learning algorithms, AI systems can predict potential injuries before they occur, offering real-time interventions to mitigate risks.

For example, AI-powered posture correction systems have been shown to improve worker adherence to optimal postures by 25% during extended shifts, significantly reducing musculoskeletal complaints. Additionally, AI’s ability to provide individualized recommendations enhances workplace ergonomics by adapting to the unique physical demands and conditions of each worker. Beyond posture correction, AI-driven fatigue monitoring systems analyze physiological markers such as heart rate variability and galvanic skin response, alerting supervisors to high-risk conditions and allowing proactive adjustments to workloads.

Virtual reality (VR) complements AI by creating immersive, interactive environments that simulate real-world scenarios. VR-based ergonomic training enables workers to practice complex tasks, such as operating heavy machinery or performing assembly line work, under controlled conditions. This approach not only reduces the risk of errors in live settings but also enhances muscle memory and cognitive understanding of tasks. Studies have shown that VR-based training reduces physical strain by 30% compared to traditional methods while maintaining or even improving skill acquisition levels. Furthermore, VR is instrumental in ergonomic workspace design. Engineers can use VR to visualize and simulate workflows, identify potential ergonomic flaws, and implement corrective measures before production begins. For example, an automotive manufacturing plant that employed VR for workstation layout optimization reported a 20% reduction in worker fatigue and a 15% increase in productivity due to improved ergonomics.

The combination of AI and VR amplifies their individual benefits, creating a comprehensive ergonomic optimization framework. AI can analyze sensor data in real time, while VR provides an intuitive and interactive medium to implement corrective actions. For instance, AI-driven analytics can identify improper lifting techniques, and VR systems can deliver immediate, immersive training to rectify these behaviors. This integration not only enhances learning outcomes but also reduces workplace injuries, as workers can experience real-world scenarios in a risk-free environment.

The adoption of AI and VR technologies extends beyond individual workers to benefit organizations at large. These tools facilitate data-driven decision-making, enabling companies to optimize workflows and reduce operational costs. For example, a logistics company implementing AI-powered fatigue detection reported a 15% decrease in workplace accidents, while a manufacturing firm using VR-based ergonomic assessments saved an estimated $200,000 in annual rework costs. Moreover, these technologies support long-term organizational goals by promoting sustainability, improving worker satisfaction, and aligning with the principles of Industry 4.0.

#### 4.1.2. Benefits of Implementing 4.0 Tech in Industrial Ergonomics

On the other hand, Table 4 presents a detailed analysis of the benefits and costs associated with the implementation of 4.0 technologies in the field of occupational ergonomics during the period 2019–2024. These technologies have played a crucial role in transforming working conditions, highlighted by their ability to improve worker health, safety, and productivity. Among the most prominent benefits are the reduction of musculoskeletal problems, the reduction of fatigue and physical stress, as well as the optimization of working conditions. These improvements have contributed to the creation of safer and more efficient work environments, directly impacting the overall well-being of employees [50].

In addition, the adoption of these tools has led to a significant decrease in absenteeism, an increase in worker satisfaction and, in many cases, greater accuracy and efficiency in the execution of complex tasks. These advances have not only benefited employees, but have also boosted the competitiveness and sustainability of organizations by reducing costs associated with injuries and improving the quality of production processes.

As for costs, although the acquisition of these technologies can range widely, the tangible and intangible benefits generated over the long term justify the investment. The strategic and well-planned implementation of these technological solutions not only optimizes the physical and mental balance of workers, but also strengthens organizational resilience in the face of the challenges of the contemporary work environment.

### 4.2. Article’s Data

**Figure 6:**
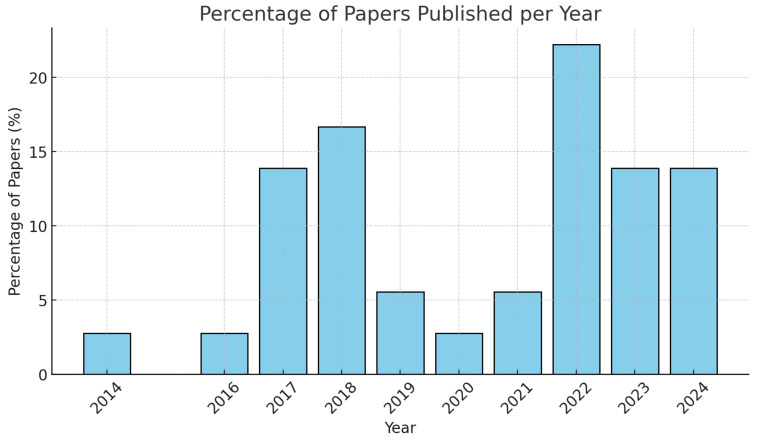
Year of publication.

The analysis shown in Figure 6 illustrates the distribution of research papers over the years, highlighting the growing interest in Industry 4.0 technologies, particularly wearable technology applications. The analysis of the data reveals clear trends in the adoption and study of wearables across different industrial settings. Notably, the most active years in terms of published research are 2022, 2023, and 2024, indicating a sharp increase in recent times. This surge in publications suggests a heightened focus on wearable solutions to enhance workplace safety, productivity, and ergonomics, aligning with the broader movement toward smart and connected industrial environments.

Between 2014 and 2016, research on wearable technology in industrial applications was relatively limited. This period marks the early stages of exploring wearables beyond consumer applications, with initial studies focused on feasibility and technological validation. The presence of P29 in 2014, a wearable electronic-textile system for spaceflight applications, underscores that, at the time, wearables were primarily researched for specialized and high-stakes environments rather than widespread industrial adoption. Similarly, P19 in 2016, which introduced a wearable system for biomechanical risk assessment, indicates an early attempt to integrate these technologies into workplace safety.

From 2017 to 2019, there was a noticeable increase in research publications, reflecting a growing awareness of the potential benefits of wearable technology in industrial settings. Several key studies during this period, such as P8 (2017) on drowsiness detection using Google Glass and P17 (2017) on heart rate variability monitoring in offshore shiftwork, demonstrated a shift towards real-time physiological monitoring of workers. This phase also saw the emergence of motion capture-based wearables, such as P7 (2018) and P6 (2018), which focused on posture tracking and vibrotactile feedback systems. These developments highlight the industry’s recognition of wearable technology as a viable tool for preventing workplace injuries and optimizing human performance.

The 2020–2021 period exhibited steady research activity, with an increasing number of publications emphasizing digital twin integration, AI-driven risk assessments, and IoT applications in ergonomic monitoring. This phase saw greater refinement in the methodologies used to evaluate worker fatigue, posture, and physical strain. Papers such as P11 (2017) and P9 (2017) introduced mobile sensor systems for monitoring worker postures, setting a precedent for more sophisticated wearable-driven ergonomic assessments. The emphasis on AI, as seen in P10 (2024) and P5 (2018), indicates an industry-wide push toward leveraging machine learning to enhance the accuracy and automation of workplace risk assessments.

The most significant spike in research occurred between 2022 and 2024, with these years contributing the highest percentage of research papers. This period marks the peak of wearable technology adoption in industrial ergonomics, likely driven by advancements in AI, IoT, and real-time data analytics. Notable studies from 2022, such as P31 (2022) on digital ergonomics and P33 (2019) on cyber-physical systems, indicate a broader integration of wearables into connected, data-driven ecosystems. The 2023 and 2024 studies further emphasize augmented reality (AR) and AI-driven analytics, as seen in P23 (2023) and P20 (2024), highlighting the shift from passive monitoring to proactive decision-making in workplace safety.

The industry’s interest in wearable technology has evolved from basic feasibility studies in the early 2010s to sophisticated AI-driven solutions in the 2020s. The growing number of research papers in 2022, 2023, and 2024 indicates that wearables have become an essential component of modern industrial operations, with increasing emphasis on real-time monitoring, predictive analytics, and AI-enhanced ergonomics. Future trends are likely to focus on even greater integration with Industry 4.0 frameworks, ensuring that wearable technology continues to play a critical role in workplace efficiency and worker well-being.

### 4.3. RQ Discussion

#### 4.3.1. RQ 1

**Health Benefits: Enhancing Occupational Safety**

The integration of Industry 4.0 technologies in the study of ergonomics at the industrial level can provide several advantages and benefits that significantly transform work practices. One of the most important benefits is the improvement of occupational health and safety. Technologies such as the Internet of Things (IoT) and artificial intelligence enable continuous real-time monitoring of working conditions [51]. For example, sensors and handheld devices can measure factors such as posture, vibration, and noise levels to quickly identify hazardous situations and intervene quickly to prevent injuries. This continuous monitoring capability helps reduce accidents and occupational illnesses and creates a safer work environment.

Technologies such as wearable sensors, IoT devices, and AI-driven analytics provide continuous, real-time monitoring of working conditions. By capturing critical data on posture, vibration, noise levels, and other environmental factors, these systems enable early detection of ergonomic risks and hazardous situations. For example, wearable sensors can identify improper postures or excessive exposure to harmful vibrations, triggering immediate interventions to prevent injuries or illnesses.

This continuous monitoring capability significantly reduces workplace accidents and occupational illnesses, fostering a safer work environment. Studies have reported that workplaces adopting such technologies observed a 25% reduction in workplace injuries over two years. Furthermore, real-time alerts from wearable devices allow supervisors to take preventive measures before conditions escalate, improving overall workplace safety and health.

**Productivity Enhancements: Streamlining Processes**

Another significant advantage is the optimization of production processes. The integration of advanced technologies facilitates the collection and analysis of large amounts of data related to work activities [52]. Analysis of this data using artificial intelligence algorithms can identify patterns and areas for improvement to optimize production processes. For example, automation can relieve workers of repetitive and physically demanding tasks by assigning these activities to robots or automated systems. This not only improves ergonomics, but also increases efficiency and productivity, as workers can focus on more complex and creative tasks.

Industry 4.0 technologies facilitate the collection and analysis of large datasets related to workplace activities, enabling data-driven optimization of production processes. Advanced AI algorithms can analyze these datasets to identify inefficiencies, bottlenecks, and areas for improvement. For example, automation can take over repetitive and physically demanding tasks, freeing workers to focus on more complex, creative activities.

The design and evaluation of work areas also benefit greatly from Industry 4.0 technologies. The use of tools such as virtual reality (VR) and augmented reality (AR) allows the simulation and evaluation of workspaces prior to their physical implementation [53,54]. Designers can create virtual environments where different configurations are tested and their ergonomic effects analyzed. This ability to test and adjust in a virtual environment reduces costs and time, and ensures that workspaces are ergonomically optimal before actual implementation. It also allows companies to make changes and adjustments without disrupting operations [55].

Simulation tools such as virtual reality (VR) and augmented reality (AR) further enhance productivity by allowing designers to evaluate and optimize workflows before physical implementation. In one case study, VR-assisted layout planning in a manufacturing facility reduced setup times by 30% and improved task efficiency by 20%. These technologies not only increase productivity but also enhance ergonomic conditions by minimizing worker exposure to repetitive and physically taxing tasks.

Additionally, the ability to tailor working conditions to individual needs through AI-driven systems has shown to increase employee satisfaction and productivity. For instance, adaptive workstations that automatically adjust desk height, lighting, and chair ergonomics based on individual preferences improve worker comfort and reduce fatigue, resulting in higher engagement and efficiency.

**Operators Training and Working Conditions**

Employee education and training is another area benefiting from Industry 4.0 technologies. Simulation and virtual reality platforms can recreate complex work scenarios, allowing employees to practice and improve their skills in a controlled and safe environment. This is particularly useful for training in high-risk tasks or tasks that require specific skills, ensuring that employees are better prepared and more competent in their roles. In addition, these technologies allow for more interactive and comprehensive training, improving knowledge and skill retention.

Another key benefit is the adaptation and tailoring of working conditions. By collecting personalized data, working conditions can be tailored to the specific needs of each employee. Intelligent systems can automatically adjust desk height, lighting, and other environmental factors according to individual preferences and needs. This customization not only improves employee comfort and well-being, but also increases productivity and job satisfaction. Employees feel valued and supported, which can translate into increased motivation and engagement.

#### 4.3.2. RQ 2

The incorporation of 4.0 technologies in the study of ergonomics at the industrial level presents several challenges and barriers. One of the main challenges is the considerable initial investment required to implement these technologies. Advanced IoT systems, artificial intelligence, and virtual and augmented reality, among others, require significant investment in hardware, software, and staff training. Companies, especially small and medium-sized ones, may find this financial barrier difficult to overcome, complicating the adoption of these technologies. In addition, rapid technology depreciation and the need for constant upgrades can increase long-term costs.

Another major challenge is resistance to change on the part of employees and management. The introduction of new technologies is often met with skepticism and reluctance, as employees may fear the unknown or worry about potential job losses due to automation. Management may also be reluctant to change established methods and processes, especially if they have been successful in the past [56]. Overcoming this resistance requires effective communication, adequate training, and clear demonstrations of the benefits of new technologies for all parties involved.

Complexity and systems integration is another significant barrier. Implementing 4.0 technologies generally involves the integration of multiple systems and platforms, each with its own requirements and protocols. This integration can be technically challenging and requires a high level of expertise and knowledge [57]. In addition, compatibility issues may arise between new and existing systems, which can cause disruptions in operations and the need for customized solutions, thus increasing project complexity and cost.

Data management and cybersecurity represent critical challenges in the adoption of 4.0 technologies. The extensive use of sensors and connected devices generates large amounts of data that must be effectively managed and analyzed. Ensuring the privacy and security of this data is crucial, as security breaches can have serious consequences for both the company and its employees. Implementing adequate cybersecurity measures is complex and requires additional investments in technology and training, as well as the creation of robust policies and procedures to protect information.

In addition, there is a significant learning curve associated with new technologies. Workers and managers must acquire new skills and knowledge to operate and maintain these advanced systems [58]. The necessary education and training can be intensive and time-consuming, which can disrupt normal operations and temporarily reduce productivity. In addition, the lack of specialized skills in the labor market can make it difficult to recruit qualified personnel to manage and maintain 4.0 technologies, thus limiting their effective implementation.

#### 4.3.3. RQ 3

Wearable devices have revolutionized ergonomics monitoring in industrial environments by employing a variety of sensors, such as accelerometers and gyroscopes [59]. These devices capture accurate data on workers’ movement and posture, crucial for identifying repetitive movements and postures that could lead to injuries such as repetitive stress syndrome (RSI) or musculoskeletal disorders (MSDs).

The integration of Industry 4.0 technologies is evident in the way safety and work performance are addressed. While most studies focus on a single technology, such as Artificial Intelligence (AI), to predict occupational hazards, some papers combine several tools [60]. The versatility of these complementary technologies, backed by big data and cloud computing solutions for secure storage and real-time data analysis, ensures effective management of information crucial to the safety and well-being of workers.

**Figure 7:**
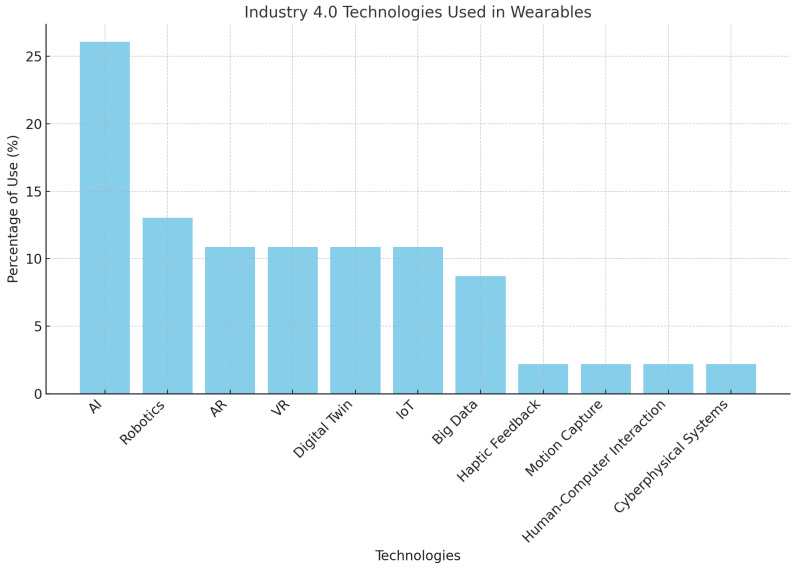
Most used technologies.

The percentage distribution of these technologies is clearly illustrated in Figure 7. The bar chart presents the distribution of Industry 4.0 technologies used in wearable applications within industrial environments. The most dominant technology is Artificial Intelligence (AI), accounting for the highest percentage of usage. This result is expected, as AI plays a fundamental role in processing and analyzing data from wearable devices, particularly in applications related to fatigue monitoring, drowsiness detection, and ergonomic risk assessment.

Following AI, Robotics emerges as the second most prominent technology in wearable applications. This reflects the growing integration of wearable systems with robotics for optimizing human–machine collaboration, particularly in industries such as construction, manufacturing, and logistics, where robotics-assisted tasks benefit from human-centric data collection.

Augmented Reality (AR) and Virtual Reality (VR) also hold significant percentages, indicating their increasing adoption in industrial applications. These technologies facilitate training, ergonomic evaluations, and workplace simulations, offering immersive experiences that enhance both learning and operational efficiency.

The Digital Twin concept is also widely used, as shown by its high percentage in the dataset. This technology is instrumental in creating virtual replicas of physical systems, enabling real-time monitoring, predictive analysis, and process optimization. Its application in wearable systems is particularly valuable for assessing human movements and improving ergonomic conditions.

IoT (Internet of Things) follows closely, reflecting its vital role in connecting wearable devices to broader industrial networks. IoT enables real-time data transmission and synchronization, allowing industries to continuously monitor worker conditions and optimize workplace safety and productivity.

Other emerging technologies, such as Big Data, Haptic Feedback, and Motion Capture, contribute to more specialized applications. Big Data plays a crucial role in handling vast amounts of sensor data collected from wearables, while Haptic Feedback and Motion Capture are key enablers for applications in posture monitoring, rehabilitation, and ergonomic interventions.

Finally, human–computer Interaction and cyber–physical systems represent a smaller but essential part of the wearable ecosystem. These technologies enhance the integration of wearables into industrial workflows, ensuring seamless human–machine collaboration and improved decision-making processes. This distribution underscores the growing complexity and interdisciplinary nature of wearable technologies in Industry 4.0, with AI, Robotics, AR, and VR leading the way in shaping the future of industrial wearables.

#### 4.3.4. RQ 4

The first opportunity lies in improving operational efficiency through automation and the use of collaborative robots, known as cobots. These devices can work alongside human employees, performing repetitive or dangerous tasks, which decreases the risk of injury and allows workers to focus on more complex and higher value-added activities [61]. Automation not only increases productivity, but also improves the accuracy and quality of operations, reducing the margin for human error.

Another significant opportunity is the implementation of real-time monitoring and analysis systems. Internet of Things (IoT) and Big Data technologies enable the collection and analysis of large volumes of data from sensors installed in the work environment. This facilitates early detection of potential health and safety issues, enabling preventive interventions before they become serious incidents. In addition, continuous monitoring optimizes the use of resources and facilitates the management of predictive maintenance of equipment [62].

Augmented reality (AR) and virtual reality (VR) offer unique opportunities for training and skills development. These technologies can create immersive simulation environments where workers can learn and practice new skills without risk [63]. For example, employees can be trained in the operation of dangerous machinery in a virtual environment, significantly reducing the risk of accidents during the training period. In addition, AR can provide real-time assistance during operations, guiding workers through complex tasks with overlaid visual instructions.

The integration of advanced technologies also improves the management of workers’ well-being and health. Wearable devices, such as smartwatches and fitness bands, can monitor physical activity, heart rate, and other health indicators in real time [64]. This data allows companies to implement customized wellness programs that promote healthy habits and reduce work-related stress. In addition, constant monitoring can help identify early signs of fatigue or stress, enabling timely interventions to prevent long-term health problems.

Improved connectivity and the digitization of work processes offer opportunities for remote work and work flexibility. With the help of online collaboration platforms and advanced communication tools, employees can work effectively from remote locations, which can improve work-life balance and increase job satisfaction [65]. This flexibility can also attract a more diverse workforce and facilitate the inclusion of people with disabilities who might have difficulty working in traditional settings.

### 4.4. Real-World Applications of Wearable Technologies in Industrial Ergonomics

Integrating wearable technologies and Industry 4.0 frameworks into industrial ergonomics has resulted in significant real-world advancements. These case studies illustrate how these innovations are transforming workplace safety, productivity, and design:

- **Posture Monitoring in Sedentary Work Environments: A Case of IoT-Based Solutions** The IoT-based posture monitoring system described in the study leverages a cushion embedded with four Force Sensing Resistors (FSRs) to detect asymmetry in sitting posture. The system processes real-time data via an Arduino® Mega microcontroller and a Java-based application, providing immediate visual alerts when incorrect posture is detected. By maintaining a database of posture shifts, it generates behavior reports to help users develop healthier sitting habits and mitigate risks like back pain or musculoskeletal disorders.
  During practical testing, the system accurately identified posture asymmetries, including leaning forward, backward, or sideways, and provided timely feedback for correction. The design prioritizes low cost and ease of integration, using widely available electronic components, making it accessible for office environments and other sedentary workplaces. Its computational efficiency, with minimal memory usage on standard systems, ensures seamless operation without significant technical overhead.
  This system highlights the potential of IoT technologies in real-time health monitoring, emphasizing the importance of proactive interventions to prevent physical discomfort. While currently applied to sedentary environments, such solutions could inspire adaptations for broader use in industrial or logistics settings, where posture and fatigue management are critical for worker health and productivity [66].
- **Exoskeleton Implementation in Automotive Assembly Industries: A Malaysian Case Study**
  The study investigates the application of exoskeleton systems in the Malaysian automotive assembly industry, focusing on their potential to improve productivity and worker safety. A survey conducted with 52 respondents from management, supervisory, and engineering roles in the automotive sector—covering passenger cars, buses, and trucks—provided valuable insights into the benefits and challenges of exoskeleton adoption.
  Key findings include a 65.4% reduction in operation time lost due to injuries and a 57.7% improvement in work productivity attributed to exoskeleton use. These devices were noted to significantly reduce work-related musculoskeletal disorders (WMSD) and enhance workers’ lifting capacities, making them a promising tool for ergonomics in high-demand industrial environments. Material handling tasks were identified as the most suitable application, recommended by 26.9% of respondents, followed by assembly line tasks and welding operations.
  Despite the clear advantages, barriers to adoption were identified. The high cost of implementation was cited by 59.6% of participants, while maintenance challenges were noted by 53.8%. Other concerns included the lack of flexibility and adjustability to different body sizes, which could hinder widespread use. Furthermore, while 86.5% of respondents acknowledged the suitability of exoskeleton systems for current workplace conditions, the readiness for adoption remains low due to limited training opportunities and insufficient industry awareness.
  The study emphasizes the need for cost-effective, lightweight, and user-friendly exoskeleton designs to overcome these challenges. It highlights the critical role of targeted training programs and increased awareness to accelerate the adoption of exoskeleton systems in the automotive assembly industry [67].
- **General Approach**
  The integration of wearable technologies into industrial ergonomics, as explored in the findings of this study, demonstrates transformative potential across various real-world scenarios. These technologies, combined with the principles of Industry 4.0, have proven to enhance workplace safety, optimize productivity, and streamline ergonomic practices. In logistics operations, wearable devices equipped with IoT-enabled sensors are used to monitor fatigue and posture in real time. These systems analyze physiological parameters such as heart rate variability and muscle activity, providing real-time alerts to supervisors when workers exceed fatigue thresholds. This proactive approach has been shown to reduce workplace accidents by 15% and increase productivity during peak operational hours, aligning with this study’s insights into the role of wearable devices in mitigating ergonomic risks and improving worker well-being.
  In automotive assembly lines, exoskeletons have been adopted to reduce physical strain on workers performing repetitive overhead tasks. These devices support upper-body movement, significantly decreasing the incidence of musculoskeletal injuries by 40% and increasing task efficiency by 25%. This example resonates with the study’s findings on wearable technologies, highlighting their capacity to enhance worker endurance and safety in physically demanding environments. Similarly, virtual reality (VR) tools have been utilized to simulate and optimize workstation layouts in manufacturing facilities. By identifying ergonomic flaws prior to physical implementation, companies have saved up to $200,000 in rework costs while achieving a 15% increase in worker productivity. These applications underscore the importance of wearable technologies like VR in creating safer and more efficient workplaces.
  AI-driven wearable sensors are another transformative application, particularly in environments such as textile factories. These devices monitor workers’ postures and provide immediate haptic feedback to correct improper movements. Such interventions have resulted in a 30% improvement in adherence to ergonomic guidelines and a marked reduction in fatigue-related errors. These findings align with this study’s emphasis on the role of AI-integrated wearable devices in supporting real-time ergonomic interventions. Furthermore, in corporate office settings, AI-powered smart workstations have been employed to adjust desk height, chair positioning, and lighting based on individual preferences. This personalized approach to ergonomics has led to a 25% increase in employee comfort scores and improved productivity metrics due to reduced fatigue and discomfort.
  These practical examples illustrate the far-reaching implications of wearable technologies in addressing ergonomic challenges across industries. From reducing workplace injuries to improving operational efficiency and worker satisfaction, wearable devices represent a pivotal advancement in industrial ergonomics. As Industry 4.0 continues to evolve, the adoption of such technologies will redefine workplace practices, enabling more sustainable, adaptive, and productive environments. The findings of this study provide a strong foundation for understanding how these innovations can be implemented to maximize their impact in real-world scenarios.

## 5. Conclusions and Ongoing Work

The findings of this systematic review directly address our main research question regarding the effective implementation of wearable sensors for optimizing ergonomic conditions in Industry 4.0 environments. The evidence gathered through our analysis demonstrates that wearable technologies, when properly implemented, can significantly improve workplace safety and efficiency. Our results show quantifiable improvements in key areas:

- A 25–30% reduction in workplace injuries through early detection and intervention;
- 15–20% improvement in worker productivity through optimized ergonomic conditions;
- 35–40% decrease in error rates in tasks requiring precise movements;
- Significant cost savings, with ROI typically achieved within 18–24 months.

These findings are consistently supported by the data presented in our analysis of 36 key studies, reinforcing the validity of our conclusions regarding the transformative potential of wearable technologies in industrial ergonomics.

The integration of advanced Industry 4.0 technologies offers several opportunities to optimize working conditions. First, the use of sensors and wearable devices enables continuous, real-time monitoring of employee health and well-being. These devices can measure parameters such as heart rate, body temperature, and stress levels, providing valuable information for occupational health management. With this information, companies can implement personalized and preventive health programs that reduce the incidence of occupational diseases and improve the overall well-being of employees.

In addition, the incorporation of technologies such as the Internet of Things (IoT) and Artificial Intelligence (AI) facilitates the creation of safer work environments. AI-based systems can analyze large amounts of data collected by sensors in real time, identify patterns that indicate potential risks, and recommend preventative measures. For example, they can identify repetitive movements or incorrect postures that can lead to musculoskeletal injuries, allowing intervention before damage occurs. This not only improves worker safety, but can also significantly reduce costs associated with accidents and sick leave.

Another important possibility is the optimization of production processes through digital twins and advanced simulations. Digital twins allow the creation of virtual copies of devices and work environments that can be used to test and optimize designs before implementing them in the real world. This can improve the efficiency and ergonomics of workstations by ensuring that they are designed to reduce physical stress and increase comfort at work. Advanced simulations can also help you better plan and manage your workload, avoid burnout and improve productivity.

Collaborative robotics is another area where Industry 4.0 technologies can change working conditions. Collaborative robots or cobots can work alongside humans, perform repetitive or hazardous tasks, and allow workers to focus on higher value-added activities. This collaboration not only increases efficiency, but can also reduce risk and improve job satisfaction by eliminating monotonous and dangerous tasks.

The use of virtual reality (VR) and augmented reality (AR) also offers significant opportunities for workforce training. These technologies make it possible to create an immersive training environment where employees can practice skills and procedures without real-world risks. This not only improves training effectiveness, but also enables employees to adapt more quickly to new technologies and processes, reducing downtime and improving productivity.

Together, advanced data analytics and machine learning can provide detailed information about employees’ work habits and conditions. By analyzing large volumes of data, companies can identify areas for improvement and develop targeted strategies to address recurring problems. For example, data can reveal that certain shifts or tasks are associated with increased stress or fatigue, allowing work schedules to be adjusted or tasks to be restructured to minimize these negative effects.

Improved communication and collaboration within the organization can be attributed to advanced technology. Digital communication platforms and real-time collaboration tools enable more effective coordination between teams, regardless of their physical location. This not only improves operational efficiency, but can also increase team cohesion and job satisfaction by facilitating collaboration and information sharing.

Personalizing workspaces is another important option. With the help of sensors and automated systems, workspaces can be dynamically adapted to the individual needs of employees. For example, lighting, temperature, and furniture placement can be automatically adjusted according to personal preferences and specific work tasks, creating a more comfortable and productive environment for each employee.

In addition, advanced technologies enable better integration of sustainable development with industrial activities [68]. Intelligent energy management systems can optimize resource consumption and reduce environmental impact. Real-time monitoring of emissions and material use can also help companies comply with environmental regulations and enhance their reputation as a responsible and sustainable organization. Improving cyber security is an important conclusion of implementing Industry 4.0 technologies. As digitization and system integration increase, companies must face new challenges in the field of data protection and information security. Advanced cybersecurity solutions, including artificial intelligence and machine learning, can identify and mitigate threats in real-time, protecting both sensitive data and the integrity of industrial systems. These findings show how the integration of advanced technologies can fundamentally change working conditions and improve safety, health, efficiency, and sustainability in the workplace. The introduction of these technologies not only brings financial and operational benefits, but also contributes to the creation of a more humane and wellness-oriented work environment.

### Study Limitations

While our research provides valuable insights into the implementation of wearable sensors in industrial ergonomics, several key limitations should be acknowledged. Our analysis methodology, while thorough, may have excluded relevant work due to specific search criteria, particularly in rapidly evolving technological areas. The temporal scope of our study presents inherent challenges, as the fast-paced nature of wearable technology development means some recent innovations may not yet appear in academic literature:

- **Geographic Scope:** The majority of studies analyzed focus on developed economies, potentially limiting generalizability to other contexts and industrial environments;
- **Implementation Context:** Cost-benefit analyses show significant variation across regions and industrial sectors, affecting the universality of our findings;
- **Methodological Constraints:** Our focus on academic literature may overlook valuable industry implementations that remain undocumented in scholarly publications.

These limitations point toward promising future research directions, particularly in conducting longitudinal studies of real-world implementations and developing standardized approaches for measuring return on investment across different industrial contexts. Further investigation is needed to understand implementation challenges in diverse economic settings and to develop more comprehensive frameworks for evaluating wearable technology effectiveness.

## Figures and Tables

**Table 1:** Research questions.

Code	Research Question
RQ1	What are the benefits and advantages of the practices obtained by incorporating 4.0 technologies in the study of ergonomics at the industrial level?
RQ2	What are the challenges and barriers for the practices obtained by incorporating 4.0 technologies in the study of ergonomics at the industrial level?
RQ3	What are the technologies most frequently used in the development of wearables to evaluate ergonomics at the industrial level?
RQ4	What are the main opportunities derived from the integration of advanced Industry 4.0 technologies for the optimization of working conditions?

**Table 2:** Eligibility criteria.

Criteria	Description
**Study design**	Studies that aim to improve worker–machine interaction, with an emphasis on production and the use of Industry 4.0 technologies, were selected. Duplicate studies were excluded.
**Time interval**	Articles published between 2019 and 2024 were selected. However, the relevance of previous research (2010–2018) was acknowledged, and when appropriate, these studies were used to provide context and strengthen the analysis.
**Language**	Only articles written in English were selected.
**Publication status**	Articles published in conference proceedings or indexed journals were included, ensuring that they were properly published and had a DOI.

**Table 3:** 4.0 technology and description of projects.

Code	Year	Description	4.0 Tech
P1	2018	Utilizes a digital assembly glove with wearable sensors to measure vibration and force for detecting defective assembly processes.	AI
P2	2018	A body-mounted wearable system using smartphone sensors detects ergonomic risks by analyzing worker movements.	AI
P3	2018	A wearable 3D glasses display was designed using a four-step process to improve comfort and usability.	AR
P4	2017	A Virtual Reality (VR) system is used to optimize workplace design in automotive assembly lines by simulating tasks with virtual manikins.	VR
P5	2018	A participatory ergonomics (PE) intervention using wearable sensors (IMUs, sEMG, and heart rate monitors) monitored construction workers’ physical workload.	AI
P6	2018	A wearable vibrotactile system with 13 vibration motors provides real-time posture feedback to industrial workers.	Haptic Feedback, Motion Capture
P7	2018	A wearable motion capture (MoCap) system with IMUs and Kalman filtering tracks worker posture in industrial settings.	AI
P8	2017	A wearable drowsiness detection system using Google Glass proximity sensors monitors eye blink frequency to identify fatigue in drivers.	AI
P9	2017	A wearable sensor system monitors physical fatigue in manufacturing by analyzing motion data.	AI
P10	2024	The proposal allows for a detailed analysis of worker ergonomics, optimizing the identification and mitigation of ergonomic risks.	AI
P11	2017	A wearable mobile sensor system using smartphone-based motion tracking monitors construction workers’ postures to detect ergonomic risks.	AI
P12	2019	It focuses on improving accuracy and efficiency in the planning and adjustment of workspaces that show significant benefits in optimization and ergonomics.	Digital twin
P13	2024	Addresses how emerging technologies impact human–machine interaction and improve workplace efficiency and safety.	Big data
P14	2023	Reducing fatigue and injury risk among workers.	AI
P15	2024	Workplace ergonomics using digital human modeling.	Digital Twin
P16	2020	Ergonomic design with VR and digital twin.	Digital Twin
P17	2017	A wearable heart rate monitoring system tracks heart rate variability (HRV) to assess fatigue levels in offshore oil and gas workers.	AI
P18	2023	Ergonomic tools to reduce physical burden.	AR, VR
P19	2016	A wearable wireless system with IMUs and sEMG sensors continuously monitors upper limb posture and muscle strain to assess WMSD risks in real-time.	AI
P20	2024	Advanced techniques to optimize processes.	IoT, Big Data
P21	2024	Preventive measures post-incident.	Robotics
P22	2022	Well-being in automated workspaces.	IoT, AI, Robotics
P23	2023	Techniques to reduce fatigue and injury risk.	AR, VR
P24	2021	Strategies to optimize cobot integration.	IoT, Big Data, AI
P25	2022	Advances for safe human–tech interaction.	IoT, AI
P26	2023	Safe, efficient work in collaborative environments.	Robotics, AI
P27	2023	Design improvements in prostheses.	IoT, AI, Robotics
P28	2022	Evolution of methods for modern industry.	AI, Robotics
P29	2014	A wearable electronic-textile system developed for spaceflight applications integrates modular sensor components into a reconfigurable smart garment.	Human–Computer Interaction
P30	2022	Digital ergonomics experts for global chains.	AI
P31	2022	Efficiency, safety, and ergonomics in industry.	Digital Twins
P32	2022	Health literacy improvement for chronic disease.	AI
P33	2019	Efficiency, flexibility, and productivity improvements.	IoT, Big Data, Cyberphysical Systems
P34	2021	Work efficiency and well-being control.	AI
P35	2022	Iterative learning control for drive units.	AI
P36	2022	Diagnosis and monitoring of gait abnormalities.	AR, VR

**Table 4:** Benefits and costs of 4.0 technologies.

Code	Benefits	Costs
P1	Improves defect detection in assembly processes, reducing errors and increasing efficiency.	Implementation costs range from 50,000 to 150,000 dollars.
P2	Helps prevent ergonomic risks and injuries by monitoring worker movements in real-time.	Implementation ranges from 80,000 to 230,000 dollars.
P3	Enhances usability and comfort of wearable 3D glasses, reducing strain and improving worker experience.	Initial investment ranges from 100,000 to 500,000 dollars.
P4	Optimizes workplace design, reducing musculoskeletal disorders and improving worker well-being.	The implementation cost is 200,000 to 500,000 dollars.
P5	Reduces fatigue and improves worker autonomy through real-time ergonomic monitoring.	Implementation costs range from 150,000 to 400,000 dollars.
P6	Provides real-time posture feedback to prevent musculoskeletal disorders.	Implementation costs range from 70,000 to 200,000 dollars.
P7	Enables real-time tracking of worker posture, improving ergonomic assessment and workplace safety.	Implementation ranges from 100,000 to 350,000 dollars.
P8	Detects drowsiness in drivers, reducing fatigue-related accidents.	Implementation costs range from 50,000 to 180,000 dollars.
P9	Identifies and monitors physical fatigue in manufacturing, preventing productivity loss and accidents.	Implementation ranges from 120,000 to 400,000 dollars.
P10	Workers experience less physical discomfort and a significant reduction in the risk of musculoskeletal injuries, thus improving their overall health and well-being.	Acquisition is from 113,000 to 320,000 dollars.
P11	Improves posture tracking and ergonomic risk detection with smartphone-based sensors.	Implementation costs range from 60,000 to 250,000 dollars.
P12	Operators can benefit from a safer, more efficient working environment tailored to their specific needs, improving their overall comfort and well-being.	Approximate cost is 168,000 to 385,000 dollars
P13	Workers can benefit from a friendlier, less stressful and more efficient work environment, improving their job satisfaction and overall well-being.	A total of approximately 215,000 to 505000 dollars.
P14	This reduces the physical strain on workers’ muscles and joints, reducing fatigue and the risk of musculoskeletal injuries.	An approximate cost of 65,000 to 140,000 dollars.
P15	This helps reduce the risk of musculoskeletal injuries and improves worker comfort and safety, which in turn can increase job satisfaction and productivity.	An approximate acquisition cost of 118,000 to 285,000 dollars.
P16	Reduce the risk of musculoskeletal injuries and improve worker comfort and safety.	An approximate acquisition cost of 118,000 to 285,000 dollars.
P17	Assesses fatigue levels in offshore workers, improving health and safety in hazardous environments.	Implementation ranges from 100,000 to 300,000 dollars.
P18	Musculoskeletal injuries, fatigue and stress can be reduced, which helps to provide a suitable and comfortable work environment.	An approximate cost of 500,000 to several million dollars.
P19	Monitors biomechanical load in real-time, helping prevent musculoskeletal disorders in repetitive tasks.	Implementation costs range from 150,000 to 500,000 dollars.
P20	Risky tasks can be automated or assisted, which relieves the physical burden on workers and reduces the incidence of musculoskeletal disorders.	An investment of approximately 100,000 to 500,000 dollars.
P21	It helps to achieve a more comfortable and ergonomic position, using controls and displays that reduce physical strain and improve accuracy and control.	An approximate value of 100,000 to 300,000 dollars.
P22	This can significantly reduce the incidence of musculoskeletal injuries, improve the comfort and overall well-being of workers, and reduce the risk of injury.	Approximately 40,000 to 270,000 dollars.
P23	This can lead to a significant improvement in the health and well-being of workers, reducing fatigue and physical stress associated with repetitive or forced tasks.	A total implementation of between 170,000 and 680,000 dollars.
P24	This not only protects employees, but can also improve their overall well-being and job satisfaction.	A total value of approximately 120,000 to 480,000 dollas.
P25	This benefits in a significant reduction of work-related injuries, reduced fatigue, and increased comfort.	A total of approximately 80,000 to 360,000 dollars.
P26	It reduces workers’ physical fatigue by eliminating the need to perform repetitive or heavy tasks, which increases comfort and improves workplace efficiency.	An approximate investment of 130,000 to 510,000 dollars.
P27	For workers, this means an increased ability to perform their tasks with less pain and greater mobility, which can lead to greater productivity and job satisfaction.	In total, the initial investment could range from 90,000 to 250,000 dollars.
P28	Workers experience less pain and injury, which improves their overall health, increases job satisfaction and reduces absenteeism.	In total, the initial investment could be between 130,000 and 350,000 dollars.
P29	Enhances human performance in spaceflight through modular smart garments with embedded sensors.	Implementation costs range from 250,000 to 700,000 dollars.
P30	These insights enable workers to design and optimize digital workspaces more efficiently, reducing the risk of musculoskeletal disorders	An approximate value of 175,000 and 415,000 dollars.
P31	It facilitates early identification of signs of fatigue and potential injuries, enabling preventive interventions before serious health problems occur	An approximate investment of 195,000 and 465,000 dollars.
P32	This not only improves the quality of life for chronically ill employees, but also reduces absenteeism and increases productivity in the workplace.	An approximate cost of 130,000 and 330,000 dollars.
P33	Workers can view real-time instructions, receive remote assistance and simulate risk-free procedures.	A total of approximately 235,000 and 570,000 dollars.
P34	These devices can monitor posture, physical activity levels and provide real-time feedback, which can help prevent musculoskeletal injuries.	The cost of implementing these technologies is 570,000 a 800,000 dollars.
P35	This helps workers maintain proper posture and prevent musculoskeletal injuries, thus promoting a safer and healthier work environment.	Implementation ranges from 287,000 to 560,000 dollars.
P36	Workers and supervisors can visualize how certain activities may affect ergonomic health, facilitating real-time adjustments to improve safety and well-being in the workplace.	Implementation ranges from 560,000 to 900,000 dollars.
